# Six Bioactive Small Molecules from *Tremella fuciformis* with Potential for Healthy Aging

**DOI:** 10.3390/foods15173045

**Published:** 2026-08-28

**Authors:** Feifei Wang, Yuxuan Duan, Ziying Cai, Shidong Lin, Lingjun Yu, Yongbiao Zheng

**Affiliations:** College of Life Sciences, Fujian Normal University, Fuzhou 350117, China

**Keywords:** *Tremella fuciformis*, bioactive small molecules, healthy aging, functional food ingredients

## Abstract

Six representative small-molecule constituents were isolated and structurally characterized from the edible mushroom *Tremella fuciformis*: *cis*-oleic acid (**1**), 1-oleoylglycerol (**2**), cerevisterol (**3**), a cerebroside (**4**), *trans*-homoaconitic acid (**5**), and uridine (**6**). Compound **5**, a tricarboxylic acid derivative, is reported for the first time as abundantly accumulated in this fungus. Aging-relevant assays showed that 1 potently inhibited α-glucosidase (IC_50_ = 0.02 mM, surpassing acarbose) and moderately inhibited acetylcholinesterase (IC_50_ = 0.29 mM); **5** competitively inhibited alkaline phosphatase (IC_50_ = 5.47 mM) and suppressed GluN2A-containing NMDA receptor currents (IC_50_ = 0.40 mM), the first such dual report for a fungal tricarboxylic acid; and **4** inhibited tyrosinase (IC_50_ = 0.80 mM) comparably to kojic acid. HPLC confirmed meaningful recovery under mild hydro-alcoholic extraction compatible with functional food processing. These multifunctional small molecules target carbohydrate metabolism, cognition, and skin homeostasis, supporting *T. fuciformis* as a source of healthy-aging food ingredients.

## 1. Introduction

Healthy aging—defined as the preservation of metabolic, cognitive, and tissue homeostasis—has emerged as a central focus in functional food research [1]. In this context, edible fungi with dual culinary and health-promoting roles represent promising functional foods endowed with a wide array of health-promoting properties, ranging from antioxidant, anti-inflammatory, and immunomodulatory activities to neuroprotection, enabling them to mitigate neurodegeneration and preserve cognitive function via the enhancement of antioxidant defenses, suppression of neuroinflammation, and inhibition of acetylcholinesterase (AChE) activity [2,3,4,5]. *Tremella fuciformis* (white jelly fungus), widely cultivated and consumed in East Asia [6], has long been valued in traditional Chinese medicine for its “lung-moistening” and “Yin-nourishing” properties, as documented in the Compendium of Materia Medica. These traditional functions align closely with homeostatic regulation and stress resistance—core pillars of healthy aging [7,8,9]. Modern pharmacological studies have primarily focused on its high-molecular-weight polysaccharides (TFPs), which demonstrate broad bioactivities including immunomodulatory [10,11,12], antioxidant [13,14], antidiabetic [15,16], and neuroprotective effects [17,18].

Nevertheless, the intrinsic structural complexity and heterogeneity of *T. fuciformis* polysaccharides (TFPs) create major obstacles to elucidating their molecular mechanisms at the protein level [19,20,21,22]. Notably, organic extracts from *T. fuciformis* exhibit potent anti-inflammatory [23], antioxidant [24], and antibacterial activities [25], which strongly imply the existence of bioactive small-molecule compounds that act independently of polysaccharides. Collectively, this evidence demonstrates that the health-promoting properties of *T. fuciformis* cannot be ascribed merely to TFPs, highlighting an understudied category of small molecules with potential biological functions.

Unlike structurally heterogeneous macromolecular polysaccharides, small molecules possess well-defined chemical skeletons, superior bioavailability, and specific binding targets, which render them ideal research objects for deciphering clear structure–activity relationships. To date, however, relevant investigations on the small-molecule components of *T. fuciformis* remain scarce, with only tentative identifications of sterols and fatty acid derivatives reported in previous literature [26,27]. Importantly, no systematic investigation has yet evaluated small molecules against aging-relevant bioactivities, particularly alkaline phosphatase (ALP; a key regulator of age-related ectopic calcification) [28,29,30] and GluN2A-containing NMDA receptors (critical modulators of cognitive aging) [31]. Moreover, their potential to modulate carbohydrate metabolism, cognitive health, and skin homeostasis remains unexplored. This lack of systematic bioactivity profiling constrains the rational development of *T. fuciformis*-based functional food ingredients.

In this work, we isolated and structurally characterized six representative small molecules from commercially cultivated *T. fuciformis* fruiting bodies, evaluating their anti-aging bioactivities and initially clarifying their structure–activity relationships and inhibitory mechanisms. This study focuses on the major isolable small-molecule fractions rather than a full-scale metabolomic profiling of all extractives, aiming to provide a scientific basis for the development of Tremella-derived functional food ingredients.

## 2. Materials and Methods

### 2.1. Materials

The dried fruiting bodies of *T. fuciformis* were purchased from a local market in Gutian, China. Alkaline phosphatase (ALP, from *Escherichia coli*, Lot#: C16003283) was obtained from Shanghai Macklin Biochemical Co., Ltd, Shanghai, China. Dimethyl terephthalate (DMT, ≥99%, Lot#: K2401069), α-glucosidase (from Saccharomyces cerevisiae, Lot#: SLBV6748), tyrosinase (from mushroom, Lot#: J1925139), AChE (from Saccharomyces cerevisiae, Lot#: slbs4398), acarbose, huperzine A, kojic acid, and ATP-2Na were supplied by Aladdin Industrial Corporation, Shanghai, China. All organic solvents were of analytical grade (Sinopharm Chemical Reagent Co., Ltd., Shanghai, China) or HPLC grade (Zhongpu Technology (Fuzhou) Co., Ltd., Fuzhou, China). Chromatographic materials included silica gel (200–300 mesh, Qingdao Marine Chemical Co., Ltd., Qingdao, China), RP-C18 silica gel (40–63 μm, Merck, KGaA, Darmstadt, Germany), and Sephadex LH-20 (GE Healthcare Cytiva, Uppsala, Sweden).

### 2.2. Instrumentation

NMR spectra were acquired on a JEOL JNM-ECZ600R/S1 spectrometer (JEOL Ltd., Tokyo, Japan). HR-ESI-MS data were recorded on an Agilent 6545 Q-TOF mass spectrometer (Agilent Technologies, Santa Clara, CA, USA). FT-IR spectra of the dried solid samples were recorded using a Thermo Nicolet 5700 FT-IR spectrometer (Thermo Fisher Scientific, Waltham, MA, USA). Enzymatic assays were measured using a Synergy HT microplate reader (BioTek, Winooski, VT, USA). Whole-cell patch-clamp recordings were performed with a HEKA EPC-10 amplifier (HEKA Elektronik, Lambrecht, Germany) controlled by PatchMaster v2x91 software. HPLC analysis was performed on a Thermo Scientific UltiMate 3000 system (Thermo Fisher Scientific, Waltham, MA, USA) equipped with an ultraviolet (UV) detector and a UMIS11C18(2) column (250 mm × 4.6 mm, 5 μm; Thermo Scientific, Waltham, MA, USA).

### 2.3. Preparation of Organic Extracts

A total of 1.15 kg of dried *T. fuciformis fruiting* bodies was extracted with 4 L of methanol. The mixture was subjected to 30 min of ultrasonication at 400 W and 40 kHz, followed by standing at 25 ± 2 °C overnight. This procedure was repeated three times to yield the combined methanol extract (25.54 g). The extract was partitioned three times between ethyl acetate and water (1:1, *v*/*v*), affording 7.12 g of the crude ethyl acetate fraction. The remaining aqueous layer was further extracted three times with an equal volume of n-butanol, resulting in 2.77 g of the crude butanol fraction.

### 2.4. Isolation and Purification of Compounds

As outlined in Figure S1, after the crude extract of ethyl acetate was dissolved with a proper amount of methanol, two fractions, A (929.2 mg) and B (203.3 mg), were obtained from the eluent of 100% methanol by RP-C18 reversed-phase silica gel column chromatography (170 g). Fraction A was further chromatographed via Sephadex LH-20 (200 g) eluting with methanol and afforded subfraction A4 (19.8 mg). Subfraction A4 was subjected to silica gel chromatography (0.75 g) using a petroleum ether–acetone solution (100:1) to yield compound **1** (5.5 mg); increasing the ratio to 15:1, compound **2** (2.4 mg) was obtained; further elution with a 4:1 ratio gave compound **3** (2.0 mg). Fraction B was separated by column chromatography over Sephadex LH-20 (120 g) with methanol eluent and afforded subfraction B4-5 (25.0 mg). Then, B4-5 was subjected to column chromatography over silica gel (0.75 g) with the eluent of a CHCl3-CH3OH solvent gradient and afforded compound **4** (3.1 mg).

Two fractions, C (551.4 mg) and D (316.7 mg), were obtained from the crude extract of butanol by medium-pressure liquid chromatography (MPLC) over RP-C18 silica gel (170 g) using ddH_2_O as the eluent. Then, fraction D was subjected to column chromatography over Sephadex LH-20 (120 g) eluting with methanol and afforded subfraction D3 (271.7 mg). Compound **5** (14.3 mg) was subjected to column chromatography over silica gel (2.0 g) with the eluent of a CHCl_3_-CH_3_OH solvent gradient. Subfraction C7 (81.4 mg) was obtained from subfraction C by column chromatography over Sephadex LH-20 (120 g) eluting with methanol. Compound **6** (4.9 mg) was obtained from subfraction C7 by column chromatography over silica gel (1.6 g) with the eluent of a CH_2_Cl_2_-CH_3_OH solvent gradient.

From 1.15 kg of dried *T. fuciformis* fruiting bodies, 25.54 g of methanol crude extract was obtained. After liquid–liquid partition, 7.12 g of ethyl acetate, 2.77 g of n-butanol, and 15.65 g of polysaccharide-rich aqueous fractions were recovered. The final yields of purified compounds 1–6 ranged from 2.0 to 14.3 mg.

The purity of all six isolated compounds was verified by HPLC via the peak area normalization method, using the same chromatographic conditions described in Section 2.5. Purity values ranged from 95.76% to 98.76% (detailed data in Table S1), all meeting the quality requirement for subsequent in vitro bioactivity assays.

### 2.5. Quantitative Analysis Procedures for the Isolated Compounds

Quantitative analysis was performed on an HPLC system equipped with an RP-C18 column. Chromatographic separation was optimized according to the physicochemical properties of these small-molecule constituents. Briefly, compounds **1**–**3** were separated using isocratic elution with acetonitrile/water (85:15, *v*/*v*) for 50 min at 190 nm. For compounds **4**–**6**, the mobile phase consisted of (A) methanol containing 0.1% (*v*/*v*) formic acid and (B) ultrapure water containing 0.1% (*v*/*v*) formic acid. Compound **4** was separated with a 40 min gradient elution program as follows: 0–5 min, 5% A; 5–15 min, 5–30% A; 15–20 min, 30–50% A; 20–25 min, 50–70% A; 25–30 min, 70–100% A; 30–33 min, 100% A; 33–35 min, 100–5% A; 35–40 min, 5% A. Compounds **5** and **6** were analyzed by isocratic elution with 5% A (5:95, *v*/*v*, A:B) for 30 min. The detection wavelength was set at 220 nm. The flow rate was 1.0 mL/min, the column temperature was maintained at 25 °C, and the injection volume was set at 0–30 μL. Calibration curves were established by plotting peak area (y) against concentration (x) using serially diluted standard solutions; all curves showed excellent linearity (R^2^ > 0.995, Table S2). The limits of detection (LOD) and limits of quantitation (LOQs) of all analytes were determined by the blank standard deviation method, with detailed numerical results listed in Table S3. A representative chromatogram of the mixed reference standards is provided in Figure S11 to demonstrate the separation performance of the method.

For sample preparation, *T. fuciformis* dry powder was extracted in triplicate (*n* = 3) using ethanol solutions of varying concentrations (60–100%, *v*/*v*) at a solid–liquid ratio of 1:20 (g/mL). Ultrasound-assisted extraction was performed at 400 W, 40 kHz, and 25 °C for 30 min, and then the mixture was allowed to stand for static maceration at room temperature overnight. This process was repeated twice. The combined extracts were filtered, concentrated, and dried to constant weight. The residues were reconstituted in ethanol (10 mg/mL), filtered through a 0.22 μm membrane, and injected into the HPLC system. The contents of the six compounds were determined by interpolating the peak areas against the respective calibration curves.

### 2.6. Bioactivity Assays

Based on the traditional consumption patterns of *T. fuciformis* [7,32] and the structural characteristics of the isolated compounds [33,34,35], the following bioactivity assays were performed to evaluate their regulatory effects on physiological processes relevant to healthy aging. All test compounds were dissolved in methanol or DMSO according to their solubility, with final organic solvent concentrations strictly controlled within a safe range. Blank controls without target biomacromolecules were set for each concentration to correct detection signals and eliminate non-specific interference.

#### 2.6.1. α-Glucosidase Inhibition

The inhibitory activity against α-glucosidase was determined using a modified literature method [36]. Briefly, 20 μL of test compound (1 mg/mL in MeOH) was mixed with 20 μL of 10 mM p-nitrophenyl-α-D-glucopyranoside (pNPG) and 100 μL of 100 mM potassium phosphate buffer (pH 6.8) in a 96-well plate. After pre-incubation at 37 °C for 15 min, 20 μL of α-glucosidase solution (0.2 U/mL) was added. Following incubation at 37 °C for 30 min, the reaction was terminated by adding 80 μL of 0.1 M Na_2_CO_3_. Absorbance was measured at 405 nm. Acarbose (1.5 mg/mL) was used as the positive control. The percentage inhibition was calculated according to Equation (1):

(1) $$\alpha ‐\mathrm{Glucosidase}\;\mathrm{inhibition}\;=\;(1-\;\frac{A_{b}\;-\;A_{1}}{A_{i}})\;\times \;100\%$$

where A_b_ is the absorbance of the reaction system with enzyme and sample, A_1_ is the absorbance without enzyme, and A_i_ is the absorbance of the vehicle control without test compound.

#### 2.6.2. AChE Inhibition

AChE inhibition was measured using a modified Ellman method [37]. Test compounds (1 mg/mL in MeOH) were incubated with 20 μL of AChE (1 μg/mL) and 100 mM phosphate buffer (pH 7.4) at 4 °C for 20 min. Then, 100 μL of 10 mM 5,5′-dithiobis (2-nitrobenzoic acid) (DTNB) and 20 μL of 10 mM acetylthiocholine iodide (ATch) were added to initiate the reaction. After 20 min at 37 °C, absorbance was recorded at 412 nm. Huperzine A (0.125 mg/mL) served as the positive control. The inhibition rate was calculated using Equation (2):

(2) $$\mathrm{Acetylcholinesterase}\;\mathrm{inhibition}\;=\;(1\;-\;\frac{A_{c}\;-\;A_{2}}{A_{s}})\;\times \;100\%$$

where A_c_ is the absorbance of the reaction system with sample and enzyme, A_2_ is the absorbance of the blank control without enzyme, and A_s_ is the absorbance of the vehicle control without test compound.

#### 2.6.3. Tyrosinase Inhibition

Tyrosinase inhibition was assessed using L-DOPA as the substrate [38]. Test compounds (1 mg/mL in MeOH) were pre-incubated with 20 μL of 2 mM L-DOPA and 160 mM phosphate buffer (pH 6.8) at 37 °C for 10 min. The reaction was started by adding 20 μL of tyrosinase (125 U/mL) and incubating for 20 min. Absorbance was measured at 475 nm. Kojic acid (10 mM) was used as the positive control. The inhibition rate was calculated using Equation (3):

(3) $$\mathrm{Tyrosinase}\;\mathrm{inhibition}\;=\;(1\;-\;\frac{A_{d}\;-\;A_{3}}{A_{t}})\;\times \;100\%$$

where A_d_ is the absorbance of the reaction system with sample, A_3_ is the absorbance of the blank control without enzyme, and A_t_ is the absorbance of the vehicle control without test compound.

#### 2.6.4. Alkaline Phosphatase Inhibition

ALP inhibition was evaluated using p-nitrophenyl phosphate (pNPP) as the substrate via an integrated and modified method based on previous reports [39,40,41,42]. Test compounds (compound **5**; *cis*-aconitic acid; *trans*-aconitic acid; 12 mg/mL in EtOH) were mixed with 30 μL of 10 mM pNPP and 110 μL of 10 mM Tris-HCl buffer (pH 8.5). After pre-incubation at 37 °C for 10 min, 10 μL of ALP (0.3 U/mL) was added. The reaction was stopped after 10 min with 10 μL of 0.1 M NaOH. Absorbance was read at 405 nm. ATP-2Na served as the positive control. The inhibition rate was calculated using Equation (4):

(4) $$\mathrm{Alkaline}\;\mathrm{Phosphatase}\;\mathrm{inhibition}\;=\;(1\;-\;\frac{A_{e}\;-{\;A}_{4}}{A_{u}})\;\times \;100\%$$

where A_e_ is the absorbance with the sample and enzyme, A_4_ is the absorbance of the blank control without the enzyme, and A_u_ is the absorbance of the vehicle control without the test compound.

Kinetic analysis of ALP inhibition was performed using Lineweaver–Burk double-reciprocal plots according to previously reported methods with minor modifications [39,43,44]. For compound **5**, inhibition kinetics were evaluated at two concentrations (0.56 and 0.67 mM) with varying concentrations of the substrate pNPP (0.03–0.48 mM). Double-reciprocal plots (1/V versus 1/[S]) were constructed using Origin 2021 to determine the inhibition type.

#### 2.6.5. Molecular Docking

Molecular docking simulations were performed to investigate the potential interactions between the selected compounds (compound **5**, *cis*-homoaconitic acid, *cis*-aconitic acid, *trans*-aconitic acid, and positive control: ATP-2Na) and ALP. The three-dimensional structures of the compounds were obtained from the PubChem database (https://pubchem.ncbi.nlm.nih.gov/, accessed on 19 August 2026; CID: 6368399; 21158450; 643757; 444212 and 5957). The crystal structure of ALP was retrieved from the Protein Data Bank (https://www.rcsb.org, accessed on 19 August 2026) under accession 1Y6V. Molecular docking was carried out using AutoDock 4.2 and AutoDockTools (ADT) 1.5.7. The protein structure was prepared by removing water molecules and adding hydrogen atoms. Semi-flexible docking was performed using the Lamarckian genetic algorithm. The grid box was set to cover the entire protein binding region. The number of GA runs was set to 50, and the maximum number of energy evaluations was 2.5 × 10^6^. The search space for blind docking was defined as a cubic grid enclosing the entire solvent-accessible surface of the receptor (dimensions 126 × 126 × 126 Å, grid spacing 0.669 Å), so that no prior assumption about the binding site was introduced. All docking poses were ranked according to binding free energy. Conformational clustering analysis was performed using a 2.0 Å root-mean-square deviation (RMSD) threshold based on ligand heavy atoms to evaluate the stability of the predicted binding poses, and the representative conformation from the lowest-energy cluster was selected as the final optimal binding mode. Finally, PyMOL 3.1.6 and Discovery Studio 2019 were used for visualization and interaction analysis.

#### 2.6.6. GluN2A-Containing NMDA Receptor Inhibition

Whole-cell patch-clamp recordings were performed according to a previously reported recombinant NMDA receptor electrophysiological protocol with modifications [45,46]. HEK293T cells (human embryonic kidney cells, Cat# CBP60439, Cobioer Biosciences, Nanjing, China; RRID: CVCL_0063, Cellosaurus) were maintained in culture for fewer than 20 passages, and all transfection and patch-clamp experiments were performed with cells at passage 13 (P13) to ensure optimal transfection efficiency, in accordance with the manufacturer’s recommendations for Lipofectamine 3000. Cells were co-transfected with hNR1-GFP and hNR2A plasmids (1.25 μg each) using Lipofectamine 3000 (Thermo Fisher Scientific, Carlsbad, CA, USA)in Opti-MEM medium. After 24 h, cells were dissociated and seeded onto glass coverslips in 24-well plates at a density of 1 × 10^5^–3 × 10^5^ cells/well and cultured for an additional 4 h prior to recording. Whole-cell patch-clamp recordings were performed at room temperature using a HEKA EPC-10 amplifier controlled by PatchMaster software. The extracellular solution contained 140 mM NaCl, 2.5 mM KCl, 1 mM CaCl_2_, and 10 mM HEPES (pH 7.4), while the intracellular solution contained 115 mM CsF, 10 mM CsCl, 10 mM EGTA, and 10 mM HEPES (pH 7.2–7.3). Patch pipettes had a resistance of 3–4 MΩ. GFP-positive cells were selected for recording and voltage-clamped at −60 mV. Current signals were sampled at 20 kHz. A multi-barrel perfusion system was used for rapid solution exchange. NMDA receptor-mediated currents were activated using extracellular solution containing 100 μM glutamate and glycine. Compound **5** (0.039–10 mM) and *trans*-aconitic acid (0.008–5 mM) were tested as single agents and co-incubated with the extracellular solution containing 100 μM glutamate + glycine. The final concentration of DMSO was maintained at 0.3‰ (*v*/*v*) in all solutions, and memantine was used as the positive control. Steady-state currents were analyzed using Clampfit 10 software. The inhibition rate was calculated according to Equation (5):

(5) $$\mathrm{Inhibition}\;\mathrm{rate}\;=\;\frac{I_{0}\;-\;I}{I_{0}}\;\times \;100\%$$

where I_0_ is the current level before compound application, and I is the current level after compound application.

### 2.7. Data Analysis

All experiments were performed in at least three independent experiments, and data are presented as mean ± standard deviation (SD). All error bars in the figures represent SDs of three independent replicates. Statistical analyses and graph generation were performed using GraphPad Prism 10. For the content comparison of six characteristic constituents across different ethanol concentrations, statistical significance was evaluated using two-way analysis of variance (ANOVA) with ethanol concentration and compound identity as independent factors, followed by Tukey’s multiple-comparison test. For intergroup comparisons of single-factor experiments, one-way ANOVA followed by Tukey’s multiple-comparison test was applied. Differences were considered statistically significant at *p* < 0.05.

IC_50_ values were calculated by nonlinear regression analysis using a four-parameter logistic model of log concentration versus inhibition rate.

## 3. Results

### 3.1. Structural Identification

**Figure 1:**
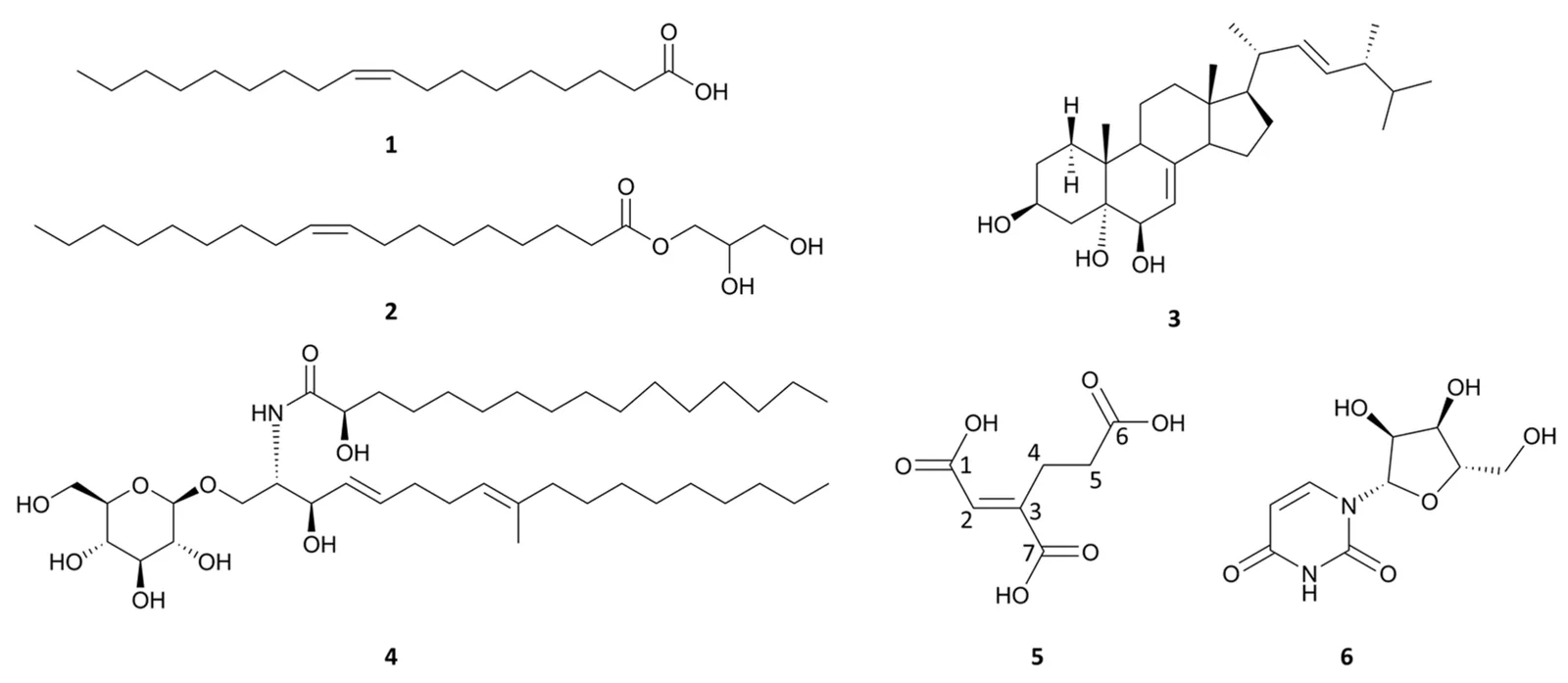
The chemical structures of compounds **1**–**6**.

Six compounds were purified from the organic extract prepared from the fruiting bodies of *T. fuciformis*, as described in the Experimental section. Their structures were elucidated by comprehensive spectroscopic analysis (^1^H NMR, ^13^C NMR, HR-ESI-MS, and FT-IR) and comparison with literature data (Figure 1).

Compounds **1**–**4** and **6** were identified as *cis*-9-octadecenoic acid (*cis*-oleic acid, Figures S2–S4) [47], 1-oleoylglycerol (Figure S5) [48], cerevisterol (Figure S6) [49], (4*E*,8*E*)-*N*-(2-hydroxypalmitoyl)-1-O-*β*-D-glucopyranosyl-9-methyl-4,8-sphingadienine (Figure S7) [50], and uridine (Figure S11) [51], respectively. Detailed spectroscopic data and full structural elucidation for these known constituents are provided in Section S1 of the Supplementary Materials.

Compound **5** was isolated as a white substance with UV (MeCN) *λ*max, nm: 222 (ε = 1075.8 L·mol^−1^·cm^−1^). The ^13^C NMR, DEPT, HSQC, and HMBC data, recorded in methanol at 151 MHz (Figure S8, Table 1), indicated that compound **5** contains 7 carbon signals: 2 methylenes at δ C 24.40 (C-4) and 34.10 (C-5); 2 *sp^2^* carbons at δ C 129.28 (C-2) and 147.06 (C-3); and 3 possible carbonyl carbons at δ C 169.49 (C-1), 176.33 (C-6) and 168.60 (C-7). Five proton peaks for ^1^H NMR (600 MHz, CD_3_OD) δ H 6.79 (d, *J* = 0.7 Hz, 1H, H-2), 3.03–2.97 (m, 2H, H-4), 2.50–2.45 (m, 2H, H-5) were observed in the ^1^H NMR spectra (600 MHz, CD_3_OD) of **5**. The molecular equation of **5** was determined to be C_7_H_8_O_6_ by HR Q-TOF MS (Figure S9) at *m*/*z* 189.0383 for [M + H]^+^ (calculated for C_7_H_9_O_6_, 189.0399), at *m/z* 211.0219 for [M + Na]^+^ (calculated for C_7_H_8_NaO_6_, 211.0219), and at *m*/*z* 187.0243 for [M − H]^−^ (calculated for C_7_H_7_O_6_, 187.0243). Based on the NMR and HR Q-TOF MS data, the planar structure of compound **5** was identified as homoaconitic acid [52]. As supplementary support for double-bond geometry, by comparison with the NMR data of *cis*-aconitic acid and *trans*-aconitic acid (Table 1), the coupling constant of the H-2 proton in compound **5** was determined to be 0.7 Hz. This value is significantly smaller than that of the corresponding proton in *cis*-aconitic acid (*J* = 1.5 Hz), and more importantly, aligns with the spectral characteristic of the H-2 proton in *trans*-aconitic acid, which appears as a singlet. Furthermore, the chemical shift of H-2 (δ 6.79) is closer to that of *trans*-aconitic acid (δ 6.91), which is quite different from that of *cis*-aconitic acid (δ 6.27). As the primary evidence for configurational assignment, Fourier-transform infrared (FT-IR) spectroscopy (Figure S10) revealed an absorption profile that diverged from that of the *cis* isomer in the carbonyl stretching (1700 cm^−1^) and fingerprint regions, consistent with the reference spectrum of synthetic *trans*-homoaconitic acid [53]. Based on the comprehensive analysis of the aforementioned NMR data and FT-IR spectroscopy data, compound **5** was identified as *trans*-homoaconitic acid. Based on the fungal α-aminoadipate lysine biosynthetic pathway, where *cis*-homoaconitic acid is the native intermediate [54], we propose that this isomer likely arises from non-enzymatic isomerization of endogenous *cis*-homoaconitic acid during purification, as pH, temperature, or solvent conditions generally favor the thermodynamically more stable *trans* configuration.

### 3.2. Quantitative Analysis Results of the Isolated Compounds

The contents of the six compounds in *T. fuciformis* (Table 2) were quantified by HPLC (Figure S12) and are reported as % *w*/*w* of dried biomass (DW). All contents are normalized to the dry weight of raw *T. fuciformis* powder, calculated from HPLC-determined concentrations in the crude extract and the corresponding extraction yield. The extraction efficiency exhibited a strong dependence on solvent polarity.

Compound **4** was the most abundant analyte, with a peak content of 0.69 ± 0.50% DW in 60% ethanol. As an amphipathic cerebroside bearing a hydrophilic glycosyl head and hydrophobic ceramide backbone, its extraction depends on balanced solvation of both moieties. Decreasing water content at higher ethanol concentrations weakens solvation of the polar sugar group, causing progressively lower yields and undetectable levels in 100% ethanol. Compounds **5** and **6** showed optimal extraction in 75% ethanol, reaching maximum yields of 0.51 ± 0.07% DW and 0.36 ± 0.02% DW, respectively. Compound **1** exhibited its highest content in 90% ethanol (0.19 ± 0.16% DW), while compound **2** was only detectable in the 90% ethanol extract with an average content of 0.04 ± 0.03% DW. Since its concentration is close to the limit of quantitation and shows large fluctuations among replicates, this value is provided only as a semi-quantitative reference for content trends. Compound **3** was tentatively identified in all crude extracts based on retention time alignment with the authentic reference standard. However, in extracts obtained with 60–80% ethanol, co-elution with co-extracted polar matrix impurities resulted in insufficient peak purity, preventing accurate peak integration. Therefore, reliable quantification was only conducted for the well-resolved peaks from the 90% ethanol (0.13 ± 0.12% DW) and 100% ethanol (0.03 ± 0.02% DW) extracts.

### 3.3. α-Glucosidase Inhibitory Activity

**Figure 2:**
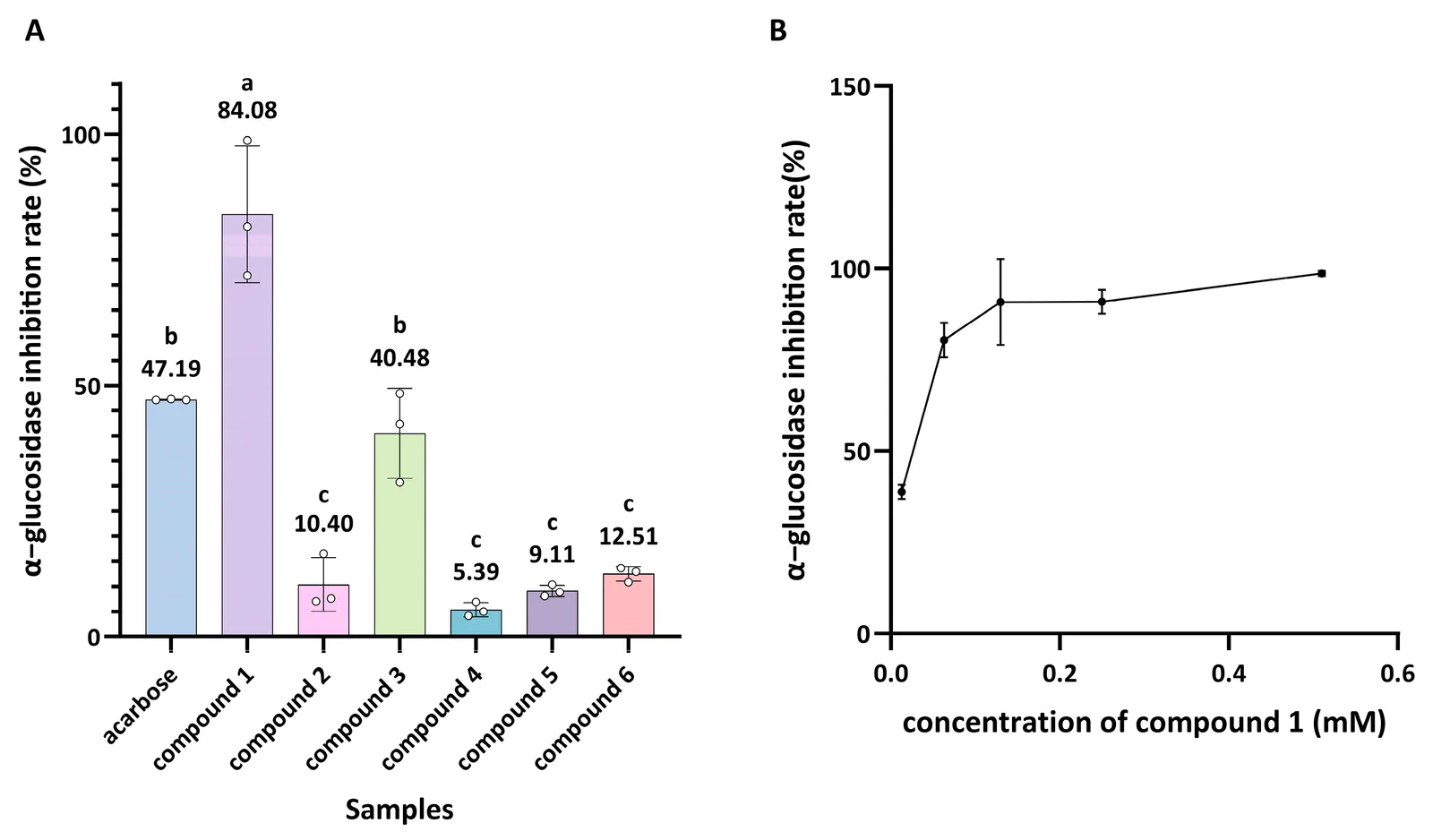
Inhibition rates of the compounds against α-glucosidase. (**A**) Inhibitory activity of different compounds against α-glucosidase. (**B**) Inhibitory activity of compound **1** at different concentrations against α-glucosidase. Data are presented as mean ± SD (*n* = 3). Different lowercase letters denote statistically significant differences among samples.

The inhibitory activities of the six isolated compounds against α-glucosidase were evaluated. In the preliminary screening, compound 1 exhibited significant inhibitory activity at 71.4 μg/mL, with stronger potency than the positive control acarbose tested at 107.1 μg/mL (Figure 2A). Compound **2** displayed only moderate inhibitory activity, while compounds **3**, **4**, **5** and **6** showed relatively weak effects. Dose-dependent analysis of compound **1** was performed over the concentration range of 3.6–142.9 μg/mL, and the results demonstrated that compound 1 inhibited α-glucosidase with an IC_50_ value of 0.02 mM (Figure 2B). This finding is consistent with previous reports describing the potent α-glucosidase inhibitory activity of cis-oleic acid with an IC_50_ value of 0.069 mM [55]. Additionally, structure–activity relationship analysis indicated that the free carboxyl group is critical for α-glucosidase inhibitory activity. Compound **1** (*cis*-oleic acid) showed markedly stronger activity than its esterified derivative compound **2** (1-oleoylglycerol). The long hydrophobic carbon chain of oleic acid may facilitate interactions with hydrophobic regions within the enzyme binding pocket, while the terminal carboxyl group participates in hydrogen-bonding interactions [56]. Esterification of the carboxyl group alters its polarity and hydrogen-bonding capacity, which weakens enzyme–ligand interactions and consequently reduces inhibitory activity. The *cis* configuration introduces a kink in the alkyl chain that may optimize spatial fitting within hydrophobic grooves, whereas trans-unsaturation or saturated analogs would adopt more extended conformations with potentially reduced complementarity. Consequently, the content of free *cis*-oleic acid, rather than its esterified forms, should be considered a key quality indicator when developing glucose-regulating functional products derived from *T. fuciformis*.

### 3.4. AChE Inhibitory Activity

**Figure 3:**
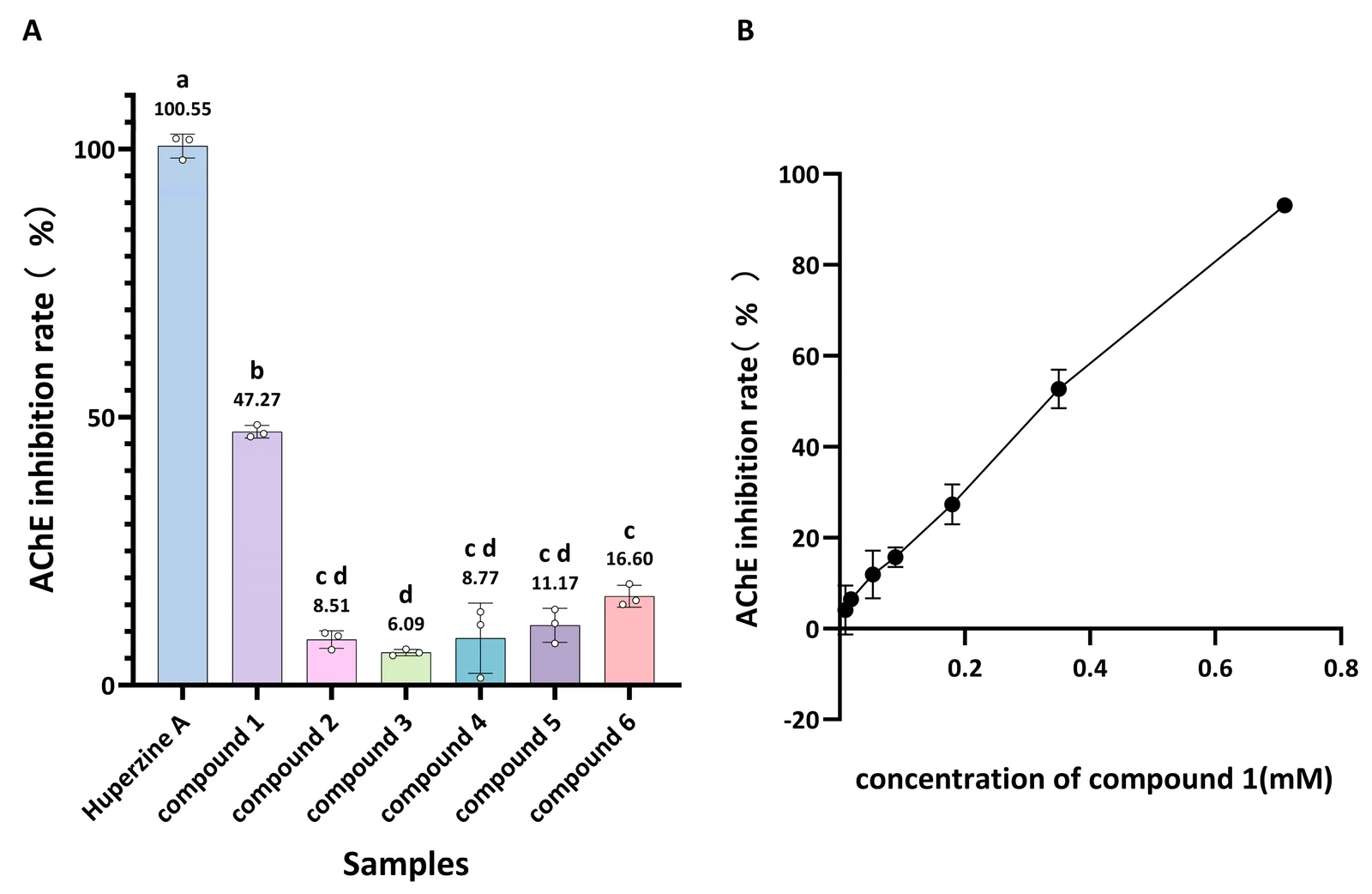
Inhibition rate of the compounds against AChE: (**A**) Inhibitory activity of different compounds against AChE. (**B**) Inhibitory activity of compound **1** at different concentrations against AChE. Data are presented as mean ± SD (*n* = 3). Different lowercase letters denote statistically significant differences among samples.

All six isolated compounds exhibited varying degrees of AChE inhibitory activity in the preliminary screening (Figure 3A). At 100 μg/mL, compound **1** exhibited the strongest inhibition (47.27 ± 1.16%), while compounds **2**–**6** showed considerably lower activities: 8.51 ± 1.65% (**2**), 6.09 ± 0.59% (**3**), 8.77 ± 6.53% (**4**), 11.17 ± 3.18% (**5**), and 16.60 ± 2.03% (**6**). Therefore, dose-dependent analysis of compound **1** was performed over a range of concentrations, and its IC_50_ value against AChE was determined to be 0.29 mM (Figure 3B). Notably, some studies have even reported that the AChE inhibitory activity of oleic acid can reach an IC_50_ of 12.01 μg/mL (0.04 mM) [57]. The structural basis for this inhibition closely parallels that observed for α-glucosidase. The marked superiority of compound **1** over compound **2** confirms that the free carboxyl group on a hydrophobic backbone is essential for potent AChE inhibition, whereas esterification abolishes hydrogen-bonding capacity and severely compromises enzyme–ligand interactions. The long hydrophobic chain and *cis*-unsaturation geometry facilitate hydrophobic interactions within the catalytic gorge, contributing to the observed inhibitory effects. Additionally, enzyme kinetic and molecular docking analyses have indicated that oleic acid inhibits AChE in a non-competitive manner by interacting with regions outside the catalytic active site. This interaction involves a hydrogen bond between the carboxyl group and Tyr334, as well as hydrophobic interactions within the catalytic gorge with residues such as Trp84, Phe290, and Phe331, which collectively contribute to the observed non-competitive inhibition pattern [35,58,59]. This non-competitive mode is pharmacologically significant, as it implies that oleic acid can modulate AChE activity without directly competing with the endogenous substrate acetylcholine, potentially offering a more nuanced regulatory effect on cholinergic neurotransmission.

### 3.5. Tyrosinase Inhibitory Activity

**Figure 4:**
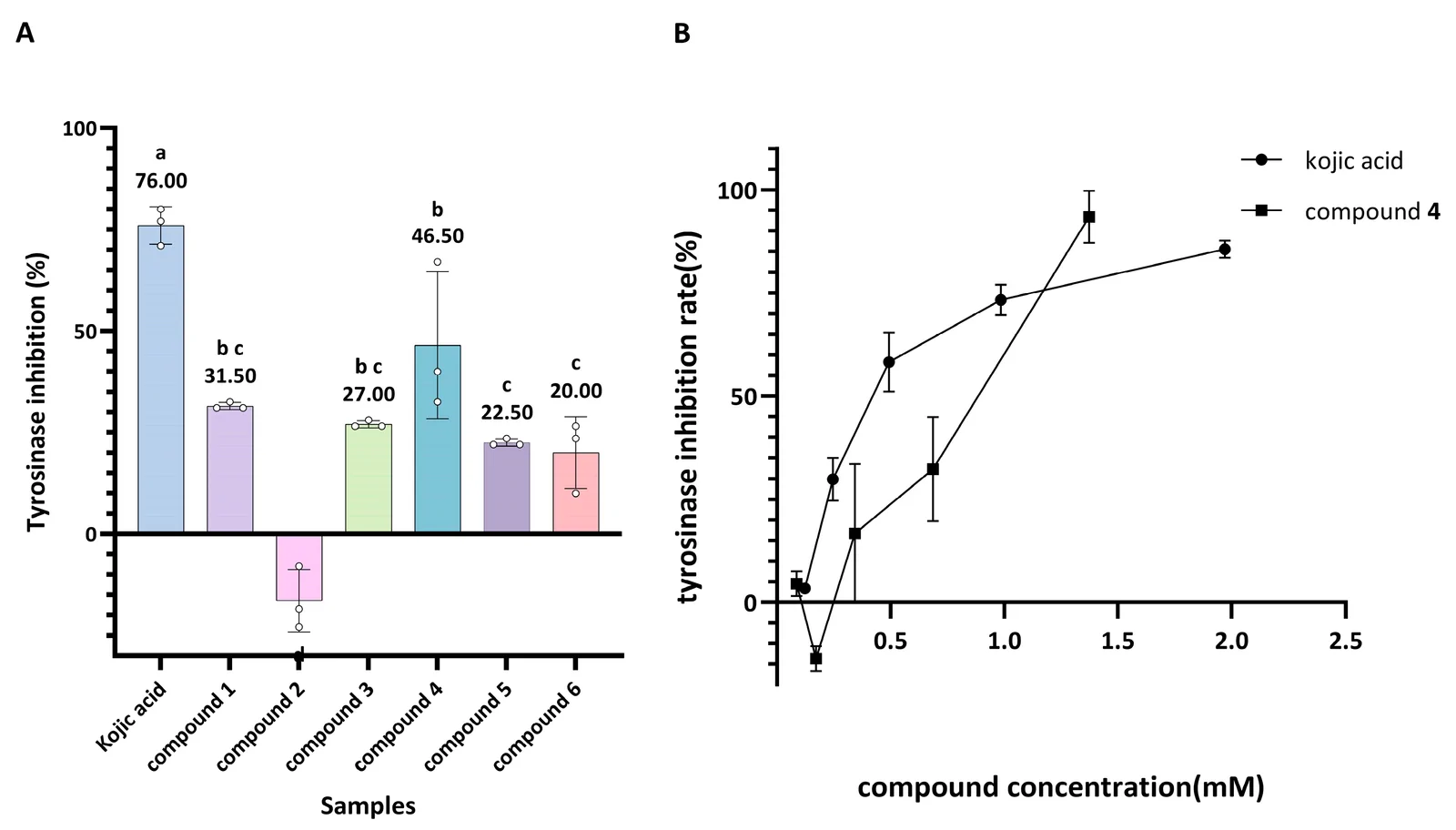
Inhibition rate of the compounds against tyrosinase: (**A**) Inhibitory activity of different compounds against tyrosinase. (**B**) Inhibitory activity of compound **4** and kojic acid at different concentrations against tyrosinase. Data are presented as mean ± SD (*n* = 3). Different lowercase letters denote statistically significant differences among samples.

All six isolated compounds were evaluated for their tyrosinase inhibitory activity. In the preliminary screening at 500 μg/mL, only compound **4** exhibited moderate inhibitory activity with an inhibition rate of 46.50%, while the remaining compounds showed either weak or negligible effects (Figure 4A). Further dose-response analysis revealed an IC_50_ value of 0.80 mM for compound **4** against tyrosinase. Although its potency is slightly lower than that of the positive control kojic acid (IC_50_ = 0.46 mM), it nonetheless demonstrates that this *T. fuciformis*-derived cerebroside possesses moderate inhibitory potential (Figure 4B). To our knowledge, this is the first report of tyrosinase inhibitory activity from a *T. fuciformis* cerebroside. Additionally, previous structure–activity relationship studies on cerebrosides have shown that the glycosylated sphingoid backbone is essential for tyrosinase inhibitory activity [60]. These studies have demonstrated that the sugar moiety introduces multiple polar hydroxyl groups capable of forming critical hydrogen-bonding interactions with key amino acid residues within the tyrosinase active site. Furthermore, the amphiphilic nature of cerebrosides facilitates more effective binding to the enzyme’s catalytic regions compared to purely hydrophobic lipids. Given that tyrosinase is the rate-limiting enzyme in melanin biosynthesis and is responsible for hyperpigmentation disorders such as melasma and age spots, this natural inhibitor offers a favorable safety profile over synthetic agents. While dedicated aggregation detection assays were not performed in the current in vitro study, this cerebroside shows preliminary research potential as a natural skin-whitening candidate for cosmetic and functional food applications targeting pigmentation control, and further validation is warranted in follow-up studies.

### 3.6. ALP Inhibitory Activity

**Figure 5:**
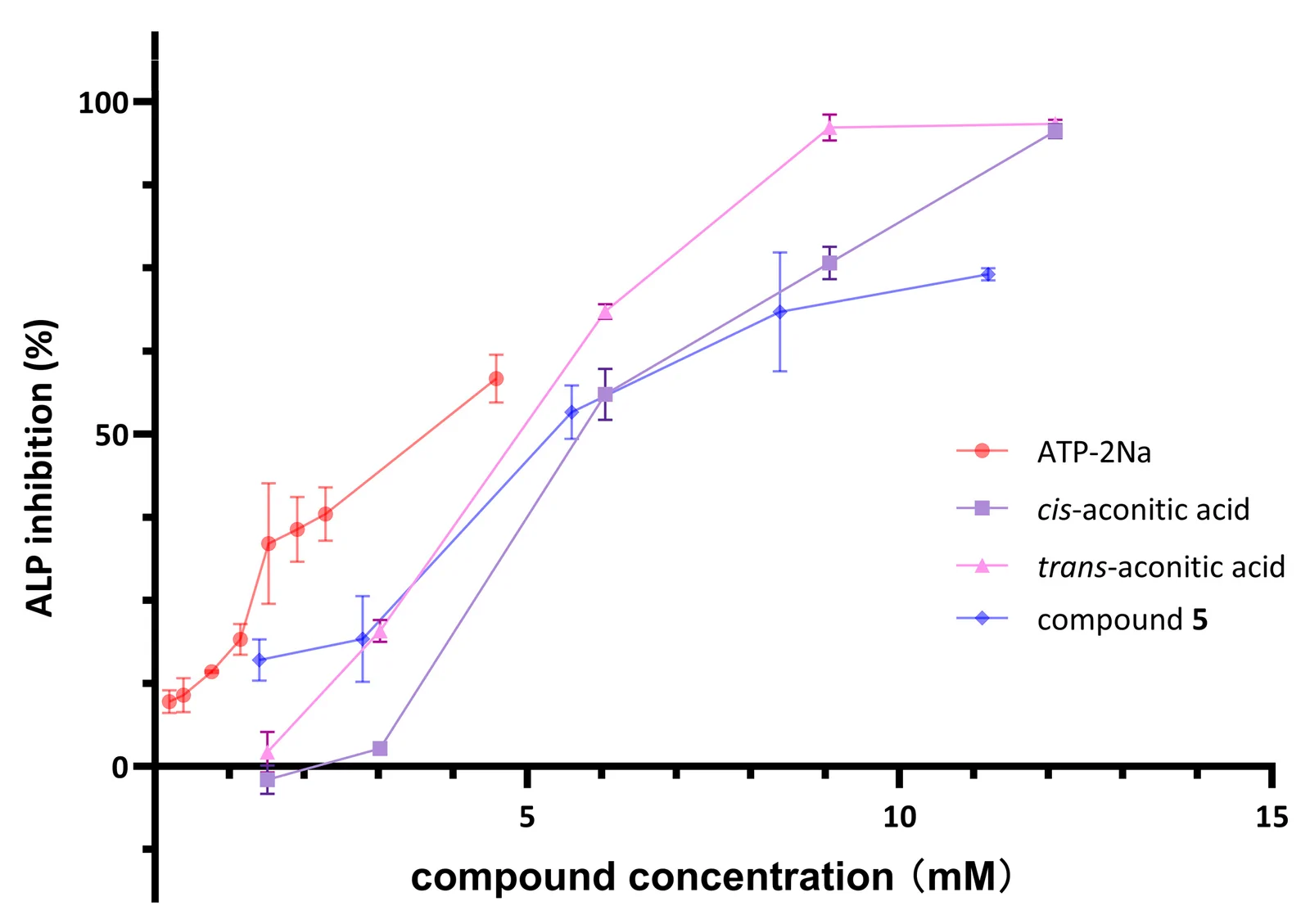
Inhibitory activity of compounds against ALP. Data are presented as mean ± SD (*n* = 3).

Alkaline phosphatase (ALP) is a metalloenzyme that plays a central role in phosphate metabolism, bone mineralization, and vascular calcification. Elevated circulating ALP is increasingly recognized as a biomarker and contributing factor in age-related metabolic, vascular, and bone disturbances, including insulin resistance, vascular calcification, and osteoporosis [28,29]. Given the high structural compatibility of the tricarboxylic acid skeleton with the negatively charged active site of ALP, compound **5** and its structurally related aconitic acid isomers (*trans*-aconitic acid, *cis*-aconitic acid) were evaluated for ALP inhibitory activity. The positive control ATP-2Na exhibited an IC_50_ value of 3.46 mM against ALP. All three experimentally tested aconitic acid derivatives showed measurable inhibitory activity, with IC_50_ values of 4.62 mM for *trans*-aconitic acid, 5.47 mM for compound **5**, and 5.96 mM for *cis*-aconitic acid (Figure 5). These findings demonstrate that aconitic acid and its derivatives represent a class of compounds with ALP inhibitory potential.

**Figure 6:**
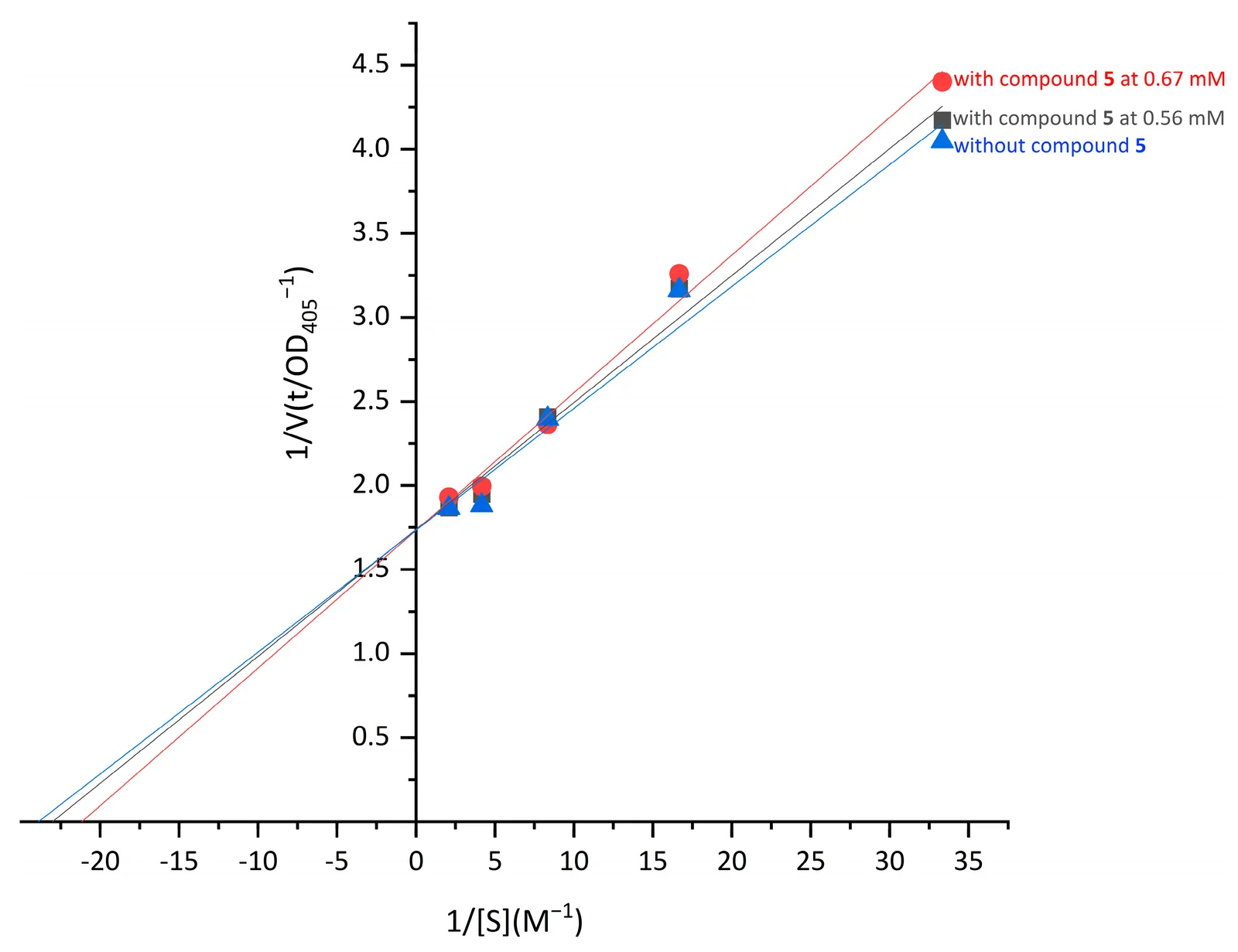
Double-reciprocal plots of the inhibition kinetics of ALP by compound **5**.

Subsequently, enzyme kinetic analysis using the Lineweaver–Burk method was performed to elucidate the inhibitory mechanism of compound **5**. As shown in Figure 6, compound **5** increased the apparent *K_m_* value without affecting the *V_max_* value, which is consistent with the characteristic pattern of competitive inhibition. This preliminary result suggests that compound **5** likely binds to the free enzyme and competes with the substrate for access to the catalytic site under the tested conditions.

**Figure 7:**
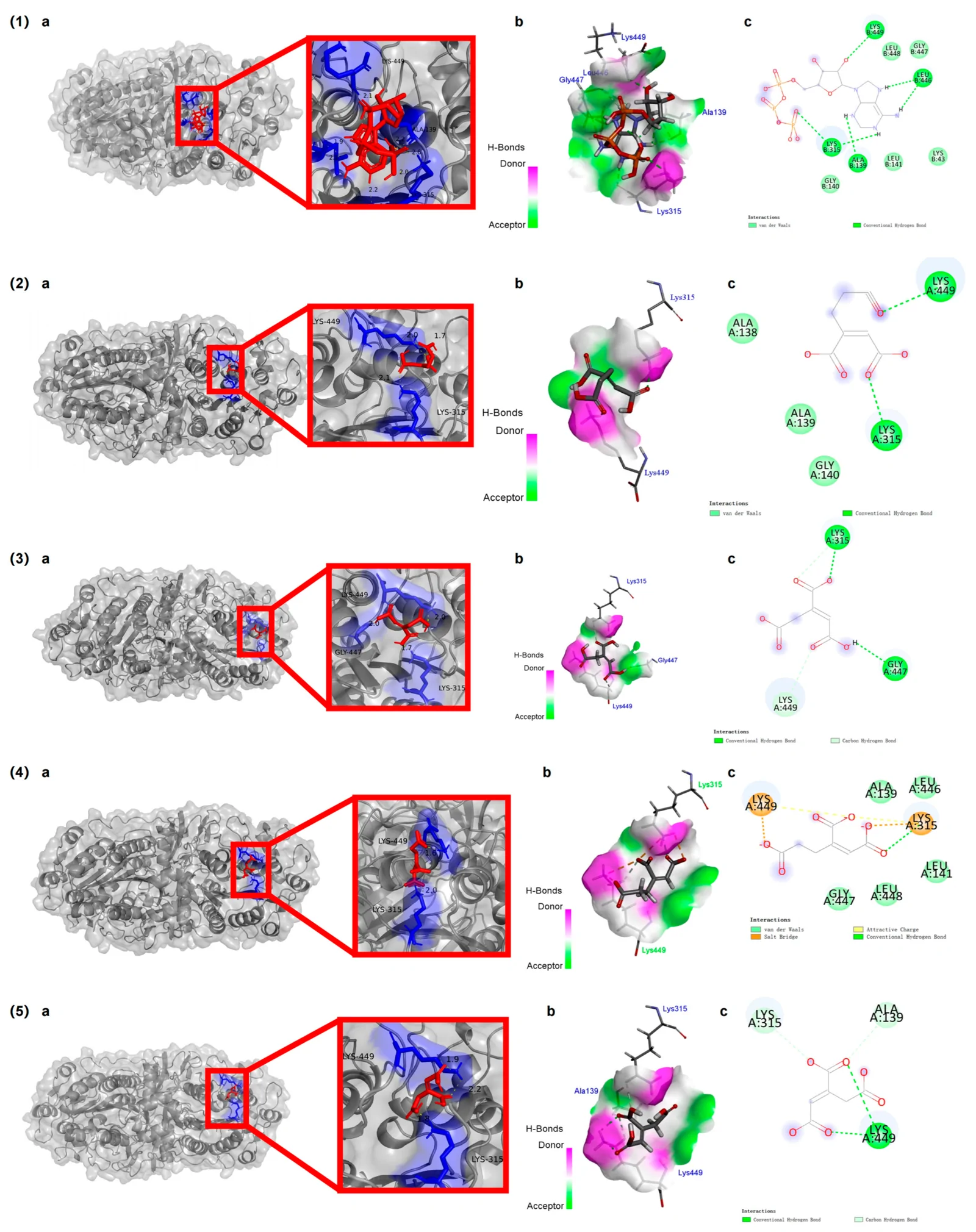
Molecular docking results of compounds with ALP: (**1**) ATP; (**2**) compound **5**; (**3**) *trans*-aconitic acid; (**4**) *cis*-homoaconitic acid; (**5**) *cis*-aconitic acid. a: 3D optimal binding poses (magnified detail in the red box); b: ligand–receptor interaction diagram; c: 2D residue interaction diagram.

Additionally, molecular docking analysis was performed to investigate the binding modes of these compounds within the ALP active site (Figure 7). Compound **5** exhibited a docking energy of −3.10 kcal/mol and formed hydrogen bonds with LYS449 and LYS315, together with van der Waals interactions involving the ALA138–GLY140 region. *Trans*-aconitic acid exhibited a binding energy of −3.32 kcal/mol, forming hydrogen bonds and C–H interactions with LYS315 and GLY447. *Cis*-aconitic acid showed a binding energy of −3.13 kcal/mol, interacting with LYS449, LYS315, and ALA139 through hydrogen bonds and van der Waals forces. Although compound **5** displayed a slightly less favorable binding energy than *cis*-aconitic acid, it exhibited stronger experimentally determined inhibitory activity, suggesting that hydrogen-bond orientation and interactions with catalytically relevant residues may contribute more substantially to inhibition than docking energy alone [61,62,63].

To expand the structure–activity relationship and explore potential superior bioactive scaffolds, *cis*-homoaconitic acid, the geometric isomer of compound **5** and the native intermediate in the fungal lysine biosynthetic α-aminoadipate (AAA) pathway, was also included in the molecular docking analysis. This compound exhibited the lowest binding energy of −4.67 kcal/mol and formed multiple hydrogen bonds, salt bridges, charge interactions, and van der Waals contacts with LYS315, LYS449, ALA139, LEU141, LEU446, GLY447, and LEU448 within the ALP active site. These extensive interactions highlight its potential as a promising lead scaffold for future optimization [64,65].

The differential binding modes and inhibitory potencies observed among these geometric isomers suggest that both geometric configuration and terminal functional group arrangement play important roles in determining ALP inhibitory activity among aconitic acid and its homo-derivatives. Consistent with this trend, *cis*-homoaconitic acid exhibited the most favorable docking energy, suggesting that the *cis* geometry may be preferable for ALP active site accommodation. Given that *cis*-homoaconitic acid represents the native chemical form in *T. fuciformis*, whereas *trans*-homoaconitic acid likely arises from isomerization during the isolation and purification process, these findings underscore the importance of preserving the native geometric configuration of key bioactive constituents during the processing of *T. fuciformis* health products. As the first report of ALP inhibitory activity for homoaconitic acid derivatives, these results expand the known bioactivity spectrum of this class of fungal tricarboxylic acids, though all experiments were conducted with *E. coli* ALP at its optimal pH of 8.5. Given the substantial structural and functional disparities between bacterial ALP and human isoforms such as bone-specific ALP, which encompass divergent physiological pH optima, direct extrapolation of these findings to human physiological conditions remains premature, and further validation with human ALP isoenzymes under physiologically relevant conditions is required in subsequent work.

### 3.7. GluN2A-Containing NMDA Receptors’ Inhibitory Activity

**Figure 8:**
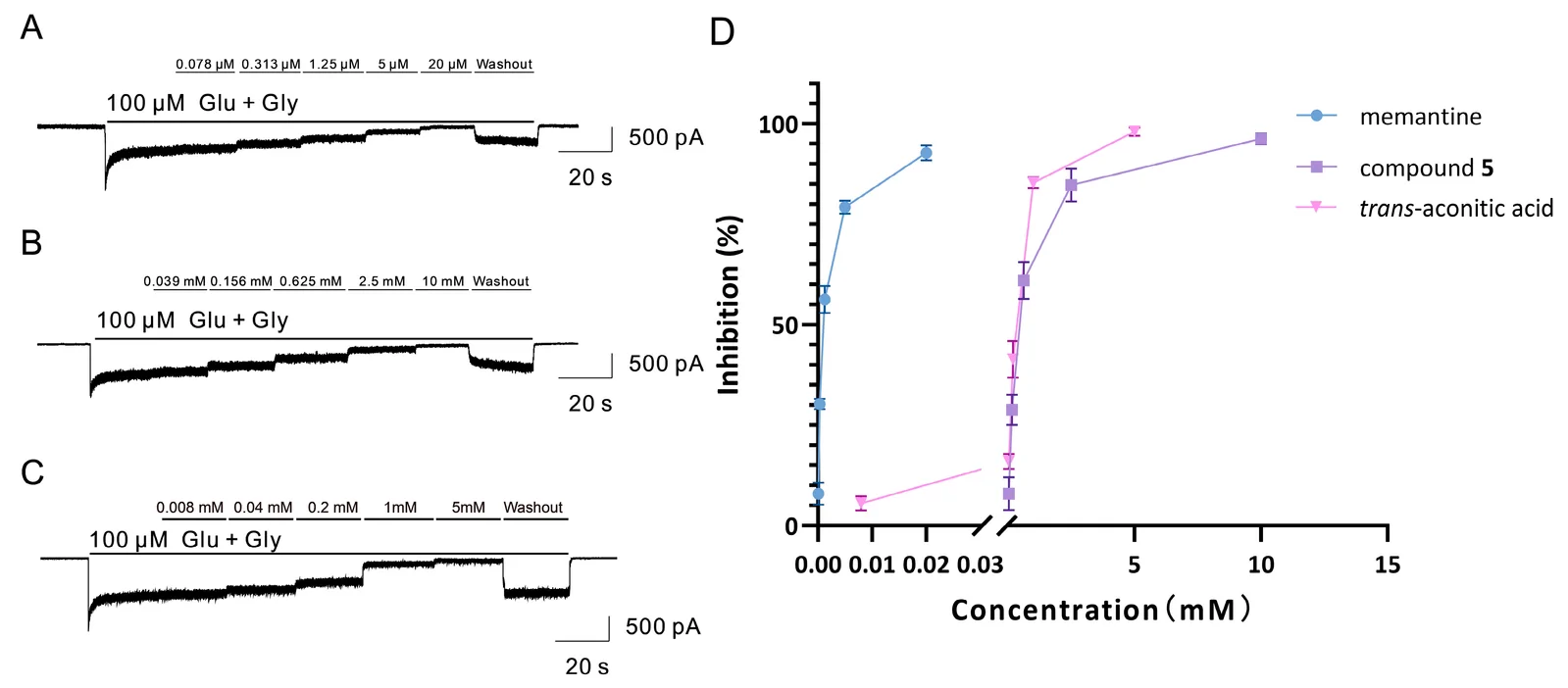
Inhibitory effects of memantine (positive control), compound **5**, and *trans*-aconitic acid on NMDA receptor currents: (**A**–**C**) Representative traces of memantine (**A**), compound **5** (**B**), and *trans*-aconitic acid (**C**) on currents induced by 100 µM Glu/Gly. Solid bars indicate cumulative drug application; rightmost sections show washout. Scale bars: 500 pA/20 s. (**D**) Concentration–inhibition curves. The *x*-axis is broken at low concentrations. Data are presented as mean ± SD (*n* = 3).

Based on the chemical structural features of compound **5** and activity predictions from SwissTarget Prediction, electrophysiological assays targeting ligand-gated ion channels were initiated. Other isolates were not included due to low structural matching. The effects of compound **5** and its lower-carbon counterpart, trans-aconitic acid, on GluN1/GluN2A-mediated currents were evaluated. Activation of the receptor with 100 μM glutamate and glycine generated sustained inward currents, which were concentration-dependently suppressed by the reference antagonist memantine (IC_50_ = 0.94 μM). Under continuous agonist perfusion, *trans*-aconitic acid suppressed the currents in a concentration-dependent manner (IC_50_ = 0.24 mM), while compound **5** also produced a progressive reduction in current amplitude with an IC_50_ of 0.40 mM (Figure 8).

A comparison shows that the inhibitory potency of the C7 analogue (**5**) is slightly attenuated relative to its C6 counterpart. This is consistent with the hypothesis that adding a single –CH_2_– modifies the molecule’s shape, surface geometry, and local hydration. Consequently, the optimal spatial complementarity, as well as the dehydration cost associated with binding at the aqueous-adjacent site, shifts modestly. This fine-tuning, rather than abolishment, preserves the suppressive activity.

The NMDA receptor, particularly the GluN2A-containing subtype, is a critical mediator of glutamatergic neurotransmission and synaptic plasticity. Excessive NMDA receptor activation and the resulting glutamatergic excitotoxicity contribute significantly to neuronal injury and cognitive decline in Alzheimer’s disease and related dementias [66,67,68]. However, many classical NMDA antagonists, including memantine, carry tolerability concerns such as psychotomimetic effects and sedation that limit their long-term use [69,70]. In this context, compound **5**, although less potent than memantine, exhibited measurable suppression of GluN1/GluN2A-mediated currents. Given its polycarboxylic structure, compound **5** may partially mimic acidic neurotransmitters, suggesting an interaction with glutamate-related binding regions [71,72]. This tricarboxylic acid scaffold from *T. fuciformis* shows both competitive enzyme inhibition (Section 3.6) and receptor modulatory activity, reflecting the broad biological effects of small-molecule constituents in this edible fungus.

To our knowledge, this is the first report of NMDA receptor current inhibition by a tricarboxylic acid derivative isolated from *T. fuciformis*. It should be noted that the effective concentration is in the millimolar range at the cellular level; potential cytotoxic effects at high millimolar concentrations cannot be ruled out without dedicated cytotoxicity assessment, and the in vivo efficacy remains to be verified. Further studies are required to elucidate the precise binding mechanism, in vivo efficacy, and pharmacokinetic properties of this compound.

### 3.8. Functional Diversity of T. fuciformis Small Molecules

The bioactivity screening revealed that the structurally diverse small molecules—ranging from unsaturated fatty acids to glycosphingolipids—exhibit distinct and complementary regulatory effects relevant to aging. Among these, *cis*-oleic acid (**1**) is a ubiquitous dietary constituent with GRAS status, yet its potent concurrent inhibition of α-glucosidase (IC_50_ = 0.02 mM) and AChE (IC_50_ = 0.29 mM) has not been systematically characterized in *T. fuciformis* previously. This bifunctional modulation bridges the epidemiological link between type 2 diabetes and Alzheimer’s disease, positioning **1** as a promising candidate for long-term nutritional strategies targeting metabolic–cognitive comorbidities. Similarly, *trans*-homoaconitic acid (**5**) represents a novel scaffold within the *T. fuciformis* metabolome. Its competitive inhibition of ALP and suppression of GluN2A-containing NMDA receptor currents (IC_50_ = 0.40 mM) are reported here for the first time. Although less potent than clinical NMDA antagonists (e.g., memantine), its low lipophilicity and natural origin suggest a favorable safety margin. However, it should be noted that *trans*-homoaconitic acid (**5**) likely arises from the isomerization of its native *cis*-counterpart during extraction and purification; nevertheless, the tricarboxylic acid scaffold itself is bioactive, as evidenced by the favorable docking energy of the native form. The cerebroside (**4**) isolated herein exhibited measurable tyrosinase inhibition (IC_50_ = 0.80 mM), offering a compelling, food-grade alternative to restricted synthetic whitening agents like hydroquinone.

These findings provide a modern molecular basis for the traditional applications of *T. fuciformis*, historically revered as the “Crown of Fungi.” The potent α-glucosidase inhibition aligns with the traditional management of “internal heat” (postprandial hyperglycemia), while AChE and tyrosinase inhibition resonate with “nourishing the brain” and skin care, respectively. Crucially, these insights shift the research paradigm away from structurally heterogeneous polysaccharides to chemically defined small molecules, establishing a framework that more precisely links traditional knowledge with contemporary scientific understanding.

As global life expectancy rises, aging-related disorders present escalating public health challenges [73,74,75]. *T. fuciformis* is uniquely positioned to address these demands due to its mature industrial cultivation; Gutian County, Fujian, China, produces over 450,000 tons annually (90% of global supply). By establishing the bioactivity profiles of specific small molecules, this study provides a scientific rationale for advancing the deep-processing industry of *T. fuciformis* toward value-added functional foods, nutraceuticals, and cosmeceuticals. Future work should prioritize in vivo validation and the elucidation of biosynthetic pathways, which will provide critical genetic targets for directed strain breeding.

## 4. Conclusions

This study isolated six major small-molecule constituents from *T. fuciformis* fruiting bodies, revealing a functional diversity that extends beyond its well-characterized polysaccharides. *Cis*-oleic acid (**1**) was identified as a multifunctional modulator of metabolic and cognitive aging, exerting concurrent inhibitory activities against α-glucosidase (IC_50_ = 0.02 mM) and AChE (IC_50_ = 0.29 mM). A cerebroside (**4**) was shown for the first time to possess tyrosinase inhibitory activity (IC_50_ = 0.80 mM), providing a novel, food-grade scaffold for skin brightening. *Trans*-homoaconitic acid (**5**) exhibited competitive ALP inhibition and suppressed GluN2A-containing NMDA receptor currents, representing the first report of such activities for this chemical class. These findings expand the scope of *T. fuciformis* research from macromolecular polysaccharides to chemically defined small-molecule constituents. Nevertheless, this work only investigated a subset of isolable small-molecule compounds from *T. fuciformis*, and the majority of low-molecular-weight extractives remain unexplored. Other uncharacterized constituents may also exhibit bioactivities relevant to healthy aging. Future studies could employ non-targeted high-resolution mass spectrometry-based metabolomics to comprehensively dissect the small-molecule metabolome of this edible fungus and further uncover its full bioactive potential. Based on the bioactive potential demonstrated by these small-molecule constituents, a promising direction for future research is to elucidate the biosynthetic pathways of these key metabolites, which could provide critical genetic targets for directed strain breeding. This would pave the way for the development of elite *T. fuciformis* cultivars enriched in specific functional ingredients, thereby accelerating the transformation of this resource into high-value functional foods, nutraceuticals, and cosmetics.

## Figures and Tables

**Table 1:** The NMR data of compound **5** (*trans*-homoaconitic acid) compared with *cis*-aconitic acid and *trans*-aconitic acid.

Position	Compound 5				*Cis*-Aconitic Acid		*Trans*-Aconitic Acid	
	^1^H	^13^C	HMBC	COSY	^1^H	^13^C	^1^H	^13^C
1		169.49 s				169.52 s		169.15 s
2	6.79 (d, *J* = 0.7 Hz, 1H)	129.28 d	C-1, C-3, C-4		6.27 (d, *J* = 1.5 Hz, 1H)	129.29 d	6.91 (s, 1H)	130.42 d
3		147.06 s				139.11 s		141.68 s
4	3.03–2.97 (m, 2H)	24.40 t	C-2, C-3, C-5, C-6, C-7	H-5	3.38 (t, *J* = 1.4 Hz, 2H)	39.98 t	3.88 (s, 2H)	33.70 d
5	2.50–2.45 (m, 2H)	34.10 t	C-3, C-4, C-6			173.23 s		173.85 s
6		176.33 s				169.17 s		168.38 s
7		168.60 s						

*Cis*-aconitic acid and *trans*-aconitic acid were included as structural analog references to confirm the double-bond geometry of compound **5** and to distinguish the homoaconitic acid skeleton from aconitic acid.

**Table 2:** Contents of compounds **1**–**6** in crude extracts obtained with different ethanol concentrations.

Compounds	60% Ethanol	70% Ethanol	75% Ethanol	80% Ethanol	90% Ethanol	100% Ethanol
**1**	0.18 ± 0.05 ^a^	0.17 ± 0.02 ^a^	0.07 ± 0.01 ^a^	0.10 ± 0.01 ^ab^	0.19 ± 0.16 ^ab^	0.06 ± 0.05 ^b^
**2**	n.d.	n.d	n.d	n.d	0.04 ± 0.03	n.d
**3**	n.d	n.d	n.d	n.d	0.13 ± 0.12	0.03 ± 0.02
**4**	0.69 ± 0.50 ^a^	0.49 ± 0.48 ^a^	0.37 ± 0.37 ^a^	0.12 ± 0.13 ^ab^	0.001 ± 0.001 ^ab^	n.d
**5**	0.26 ± 0.02 ^a^	0.37 ± 0.12 ^a^	0.51 ± 0.07 ^a^	0.29 ± 0.09 ^ab^	0.04 ± 0.01 ^ab^	0.01 ± 0.004 ^b^
**6**	0.17 ± 0.01 ^a^	0.24 ± 0.08 ^a^	0.36 ± 0.02 ^a^	0.26 ± 0.09 ^ab^	0.05 ± 0.002 ^ab^	0.01 ± 0.01 ^b^

Values are expressed as mean ± SD (*n* = 3). n.d.: not detected. Statistical analysis was performed using two-way ANOVA followed by Tukey’s post hoc test. Different lowercase letters within the same compound indicate significant differences (*p* < 0.05).

## Data Availability

The data supporting the findings of this study are available within the article and its Supplementary Materials. All NMR, MS, GC-MS, and FT-IR spectra of the isolated compounds have been provided in the Supplementary Information. No additional external datasets were generated or analyzed during this study.

## Supplementary Materials

The following supporting information can be downloaded at: https://www.mdpi.com/article/10.3390/foods15173045/s1, Figure S1: Extraction and isolation scheme of compounds **1**–**6** from *T. fuciformis*, Figure S2: The ^13^C NMR and ^1^H NMR spectra of compound **1** (A) ^13^C NMR spectrum (B) ^1^H NMR spectrum, Figure S3: FT-IR spectrum of compound **1**, Figure S4: GC–MS spectrum of compound **1**, Figure S5: The ^13^C NMR and ^1^H NMR spectra of compound **2**, (A) ^13^C NMR spectrum (B) ^1^H NMR spectrum, Figure S6: The ^13^C NMR and ^1^H NMR spectra of compound **3** (A) ^13^C NMR spectrum (B) ^1^H NMR spectrum, Figure S7: The ^13^C NMR and ^1^H NMR spectra of compound **4** (A) ^13^C NMR spectrum (B) ^1^H NMR spectrum, Figure S8: The ^13^C NMR and ^1^H NMR spectra of compound **5** (A) ^13^C NMR spectrum (B) ^1^H NMR spectrum, Figure S9: High-resolution ESI-MS spectra of compound **5** (A) Positive ion mode (B) Negative ion mode, Figure S10: FT-IR spectrum of compound **5**, Figure S11: The ^13^C NMR and ^1^H NMR spectra of compound **6**, (A) ^13^C NMR spectrum (B) ^1^H NMR spectrum, Figure S12: HPLC profiles of compounds **1**–**6** and crude extracts from different solvent extractions (A) Compounds **1**–**3** and crude extracts. (B) Compound 4 and crude extracts. (C) Compounds **5**–**6** and crude extracts, Table S1: HPLC purity of compounds **1**–**6**, Table S2: Calibration curve equations and coefficients of determination (R^2^) for compounds **1**–**6**, Table S3: LOD and LOQ for compounds **1**–**6**.
