# Supplementary material for: Six Bioactive Small Molecules from Tremella fuciformis with Potential for Healthy Aging
Source: Foods. 2026 Aug 28;15(17):3045. doi: 10.3390/foods15173045 (PMC13564794; doi:10.3390/foods15173045)
Supplement: Supplementary file 1 [file foods-15-03045-s001.zip › foods-4486240-supplementary.pdf]

Supplementary Material for *Foods*

Small Molecules from *Tremella fuciformis* with Diverse Bioactivities for Healthy Aging

Feifei Wang[1], Yuxuan Duan[1], Ziying Cai[1], Shidong Lin[1], Lingjun Yu[1*], Yongbiao Zheng[1*].

[1] College of Life Sciences, Fujian Normal University, Fuzhou, 350117, PR China

Section S1 *Detailed Spectral Data of Compounds **1**–**4** and **6***

The detailed spectroscopic data for the five known characteristic constituents are provided below as supplementary support for the structural identification described in Section 3.1 of the main text. All spectra were acquired under consistent conditions, and structural assignments were confirmed by comparison with published literature data.

Compound **1** was obtained as a white powder. Its structure was elucidated based on NMR (Figure S2), IR (Figure S3), and GC–MS (Figure S4) analyses. The IR spectrum exhibited characteristic absorptions at 3005 cm^{-1} (olefinic C–H), 2922 and 2853 cm^{-1} (aliphatic C–H), 1709 cm^{-1} (carboxyl C=O), and 722 cm^{-1} (out-of-plane bending of a cis-disubstituted C=C). The absence of a strong band near 965 cm^{-1} ruled out a trans-configuration. The ^{1}H NMR (600 MHz, $CDCl_3$) spectrum displayed olefinic proton signals at δ 5.39–5.30 (m, 2H), a terminal methyl at δ 0.88 (t, *J*= 7.0 Hz, 3H), and an allylic methylene at δ 2.01 (d, *J*= 5.3 Hz, 2H); the resolved long-range coupling (^{4}J) observed for the allylic protons further corroborated the *cis*-geometry of the Δ^9 double bond. The ^{13}C NMR (151 MHz, $CDCl_3$) spectrum revealed a carbonyl carbon at δ 179.10, olefinic carbons at δ 130.19 and 129.89, and a typical aliphatic chain envelope (δ 29.2–29.9). Comparison of these spectroscopic data with those reported in the literature confirmed compound **1** as *cis*-9-octadecenoic acid (*cis*-oleic acid) .

Compound **2** was isolated as a white oily product, and its structure was determined by ^{1}H and ^{13}C NMR spectroscopic analysis (Figure S5). The ^{1}H NMR (600 MHz, CD_3OD) spectrum showed signals at δ 5.42–5.26 (m, 2H), 4.15 (dd, *J* = 11.4, 4.4 Hz, 1H), 4.06 (dd, *J* = 11.5, 6.3 Hz, 1H), 3.81 (qd, *J* = 5.8, 4.3 Hz, 1H), 3.55 (dd, *J* = 5.6, 3.5 Hz, 2H), 2.35 (t, *J* = 7.4 Hz, 2H), 2.03 (dd, *J* = 5.8, 1.9 Hz, 2H), 1.63 (d, *J* = 6.8 Hz, 2H), 1.35–1.28 (m, 25H), 0.92–0.89 (m, 3H), and the ^{13}C NMR (151 MHz, CD_3OD) spectrum exhibited signals at δ 175.69, 130.91, 130.81, 71.18, 66.49, 64.10, 33.05, 30.83, 30.74, 30.70, 30.59, 30.43, 30.33, 30.28, 30.19, 30.17, 28.11, 25.99, 23.72, 19.45, 14.42. The NMR spectra of compound **2** retained all characteristic signals of the oleic acid skeleton present in compound **1**, including the *cis*-olefinic protons, allylic methylene, and aliphatic chain envelope, but displayed additional oxygenated proton signals at δ 3.55–4.15 and corresponding oxygenated carbon signals at δ 64.10–71.18, which were attributed to a glycerol moiety esterified to the carboxyl group of oleic acid. By comparison with previously reported literature data, compound **2** was identified as 1-oleoylglycerol.

Compound **3** was obtained as a white powder, and its structure was elucidated by comprehensive NMR spectroscopic analysis (Figure S6). The ^{1}H NMR (600 MHz, CD_3OD) spectrum showed signals at δ 5.25 (m, 1H), 5.22 (dd, *J* = 7.7, 6.7 Hz, 1H), 3.98 (tt, *J* = 11.4, 4.9 Hz, 1H), 3.55 (dt, *J* = 5.3, 2.1 Hz, 1H), 2.27 (t, *J* = 7.5 Hz, 1H), 2.13–2.07 (m, 2H), 1.90 – 1.82 (m, 1H), 1.81–1.71 (m, 2H), 1.72–1.66 (m, 1H), 1.66–1.39 (m, 6H), 1.38–1.28 (m, 7H), 1.06 (s, 3H), 1.04 (d, *J* = 6.6 Hz, 3H), 1.00 (d, *J* = 6.7 Hz, 1H), 0.94 (d, *J* = 6.8 Hz, 3H), 0.87 (d, *J* = 6.8 Hz, 3H), 0.85 (d, *J* = 6.8 Hz, 3H), 0.65 (s, 3H), and the ^{13}C NMR (151 MHz, CD_3OD) spectrum exhibited signals at δ 143.78, 137.04, 133.26, 119.09, 76.95, 74.25, 68.40, 57.42, 55.92, 44.71, 44.38, 44.36, 41.79, 40.73, 40.52, 38.16, 34.38, 33.92, 31.77, 29.15, 24.02, 23.04, 21.66, 20.46, 20.08, 19.45, 18.21, 14.42. By comparison with the reported literature data, compound **3** was identified as cerevisterol.

Compound **4** was obtained as a white powder, and its structure was elucidated by comprehensive ^{1}H and ^{13}C NMR spectroscopic analysis (Figure S7). The ^{1}H NMR (600 MHz, CD_3OD) spectrum showed signals at δ 5.78–5.69 (m, 1H), 5.48 (ddd, *J* = 15.5, 7.4, 1.5 Hz, 1H), 5.15 (tt, *J* = 5.6, 2.8 Hz, 1H), 4.27 (d, *J* = 7.7 Hz, 1H), 4.17–4.10 (m, 2H), 3.99 (m, 2H), 3.87 (dd, *J* = 11.9, 1.7 Hz, 1H), 3.74–3.64 (m, 2H), 3.38–3.33 (m, 1H), 3.30–3.25 (m, 2H), 3.19 (dd, *J* = 9.2, 7.8 Hz, 1H), 2.13–2.03 (m, 4H), 1.98 (t, *J* = 7.6 Hz, 2H), 1.71 (m, 1H), 1.60 (s, 3H), 1.55 (m, 1H, H-3b), 1.44–1.35 (m, 4H), 1.34–1.24 (m, 28H), 0.98–0.84 (m, 6H), and the ^{13}C NMR (151 MHz, CD_3OD) spectrum exhibited signals at δ 177.22, 136.79, 134.66, 131.13, 124.84, 104.74, 78.00, 77.92, 75.00, 73.10, 72.90, 71.58, 69.74, 62.68, 54.62, 40.79, 35.89, 33.80, 33.09, 30.82, 30.77, 30.70, 30.50, 30.39,

29.12, 28.69, 26.16, 23.75, 16.13, 14.46, 14.44. The NMR spectra displayed characteristic signals of a cerebroside, including an anomeric proton at δ 4.27 (d, *J* = 7.7 Hz, 1H) corresponding to a *β*-*D*-glucopyranosyl moiety, two olefinic proton signals at δ 5.78–5.48 assigned to the 4*E* and 8*E* double bonds of the sphingadienine backbone, an amide carbonyl carbon at δ 177.22, and long aliphatic chain signals. By comparison with previously reported literature data, compound **4** was unambiguously identified as (4*E*,8*E*)-*N*-(2-hydroxypalmitoyl)-1-O-*β*-*D*-glucopyranosyl-9-methyl-4,8-sphingadienine .

Compound **6** was obtained as a white powder, and its structure was elucidated by comprehensive ^{1}H and ^{13}C NMR spectroscopic analysis (Figure S11). The ^{1}H NMR (600 MHz, CD_3OD) spectrum showed signals at δ 8.01 (d, *J* = 8.1 Hz, 1H), 5.90 (d, *J* = 4.7 Hz, 1H), 5.70 (d, *J* = 8.1 Hz, 1H), 4.16 (dt, *J* = 19.2, 5.1 Hz, 2H), 4.00 (dt, *J* = 4.7, 2.9 Hz, 1H), 3.84 (dd, *J* = 12.2, 2.7 Hz, 1H), 3.73 (dd, *J* = 12.2, 3.1 Hz, 1H), and the ^{13}C NMR (151 MHz, CD_3OD) spectrum exhibited signals at δ 166.22, 152.47, 142.74, 102.63, 90.68, 86.36, 75.73, 71.30, 62.26. The NMR spectra displayed characteristic signals of a nucleoside, including two doublet pyrimidine ring protons at δ 8.01 and 5.70, an anomeric proton of ribose at δ 5.90, and multiple oxygenated carbon signals corresponding to the ribose moiety. By comparison with previously reported literature data, compound **6** was identified as uridine.

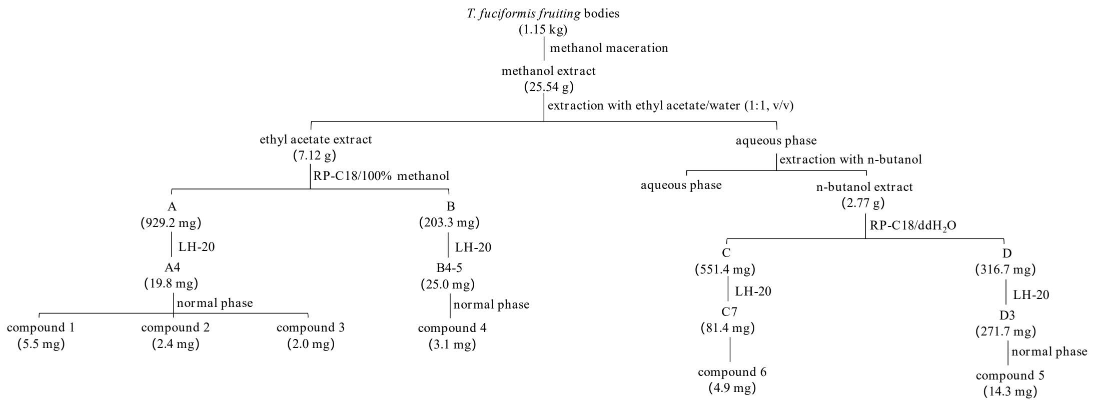

Figure S1. Extraction and isolation scheme of compounds **1–6** from *T. fuciformis*.

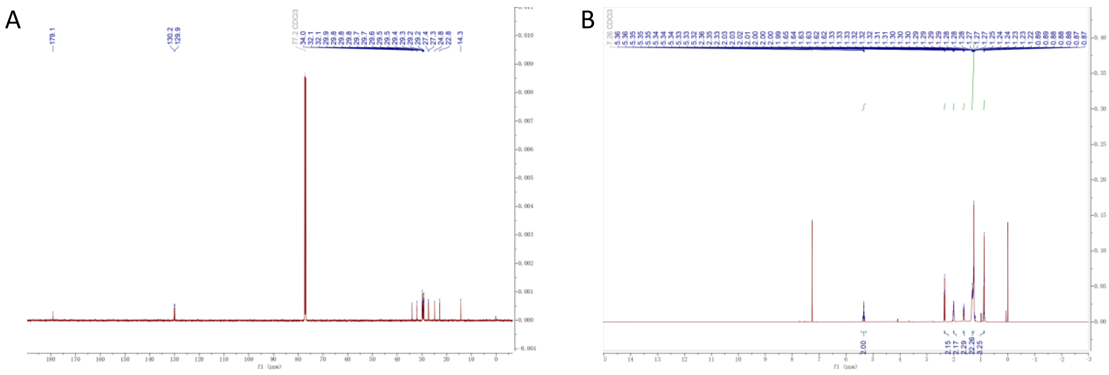

Figure S2. The ^{13}C NMR and ^{1}H NMR spectra of compound **1**. (**A**) ^{13}C NMR spectrum; (**B**) ^{1}H NMR spectrum.

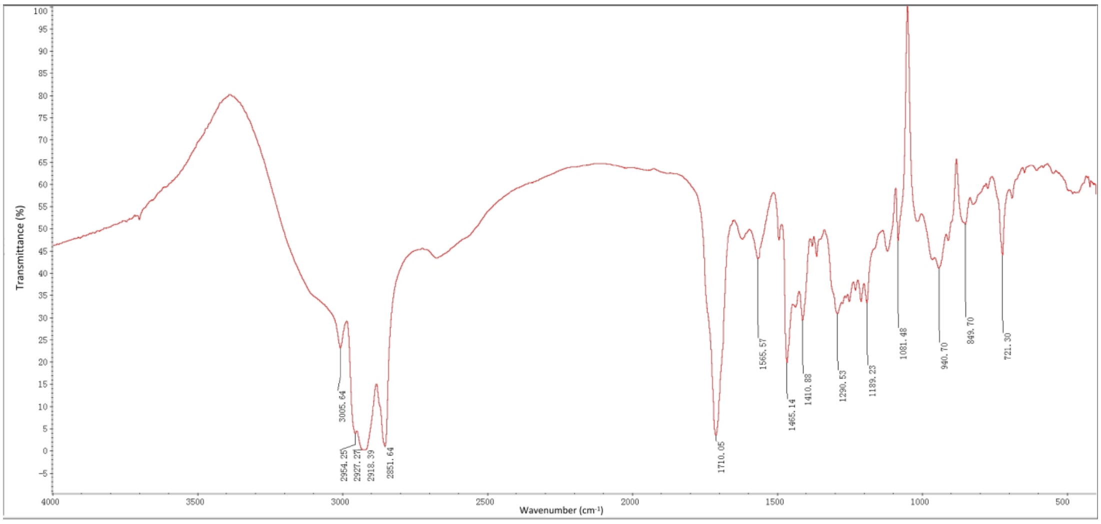

Figure S3. FT-IR spectrum of compound **1**.

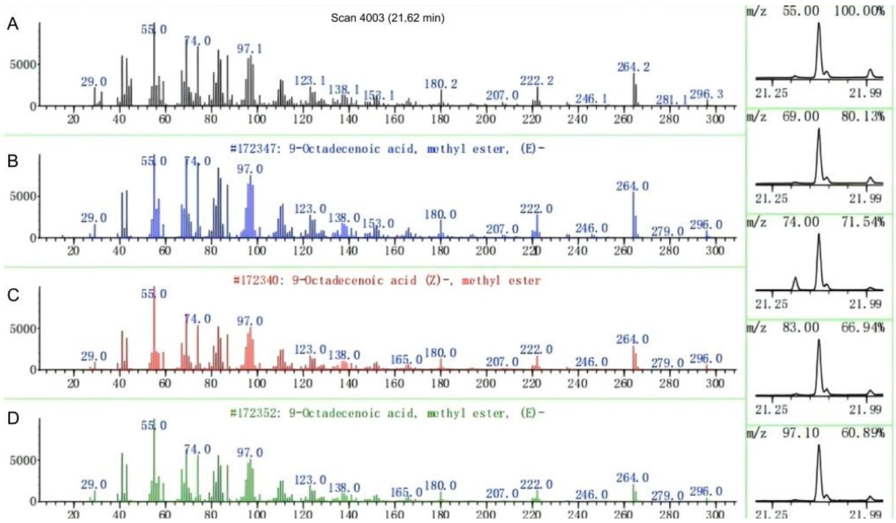

Figure S4. GC–MS spectrum of compound **1**. (A) Detected mass spectrum of the sample peak at retention time 21.62 min; (B – D) The three best matching spectra retrieved from the NIST 17.L library: (B) methyl (E)-9-octadecenoate (CAS: 1937-62-8, match quality: 99), (C) methyl (Z)-9-octadecenoate (CAS: 112-62-9, match quality: 99), (D) methyl (E)-9-octadecenoate (CAS: 1937-62-8, match quality: 99). This component accounts for 23.36% of the total peak area.

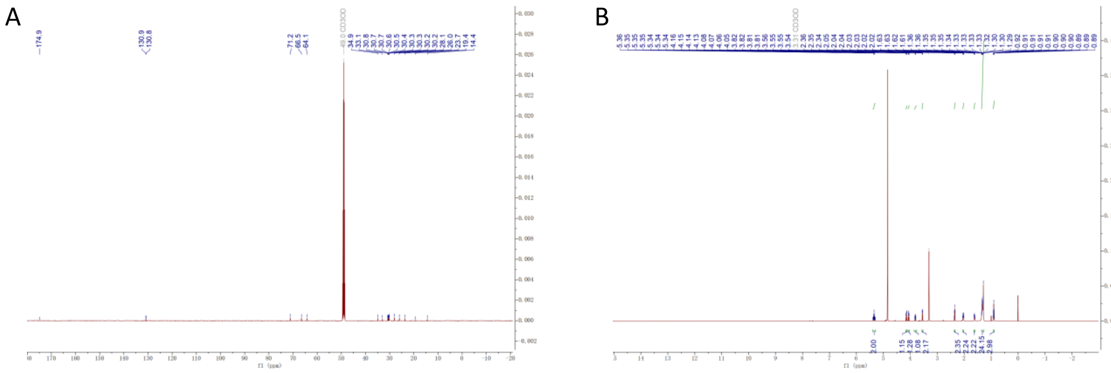

Figure S5. The ^{13}C NMR and ^{1}H NMR spectra of compound **2**. (**A**) ^{13}C NMR spectrum; (**B**) ^{1}H NMR spectrum.

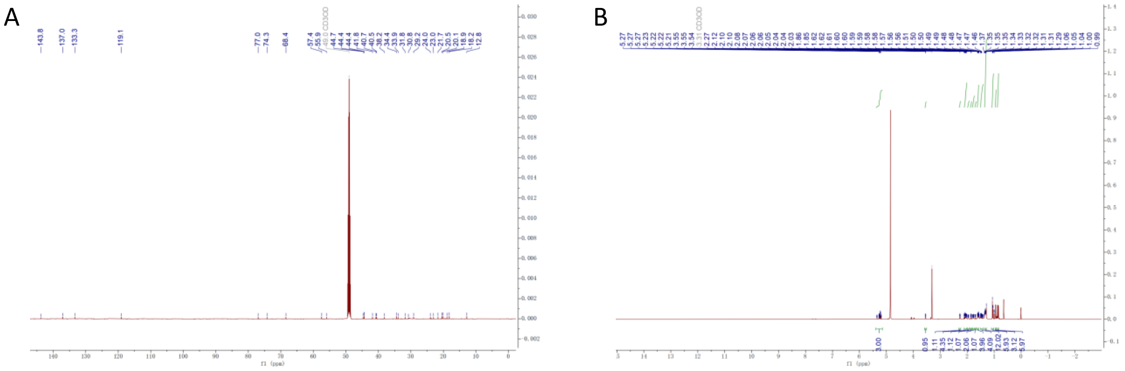

Figure S6. The ^{13}C NMR and ^{1}H NMR spectra of compound **3**. (**A**) ^{13}C NMR spectrum; (**B**) ^{1}H NMR spectrum.

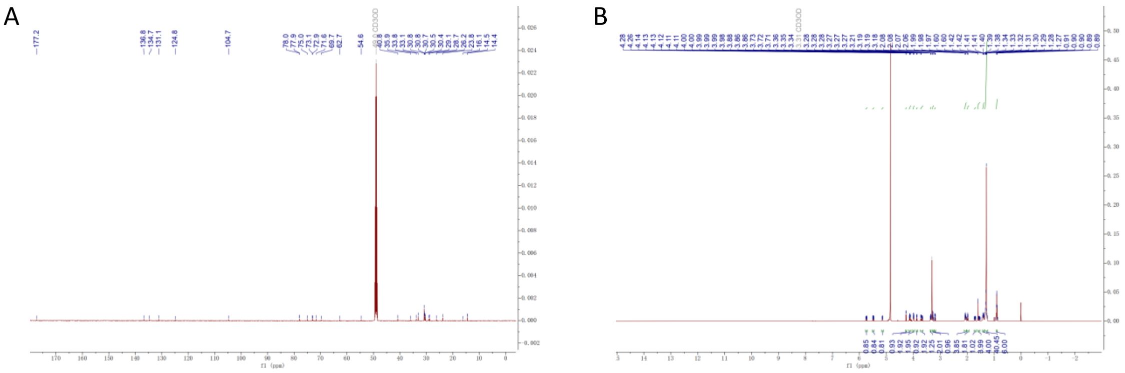

Figure S7. The ^{13}C NMR and ^{1}H NMR spectra of compound **4**. (**A**) ^{13}C NMR spectrum; (**B**) ^{1}H NMR spectrum.

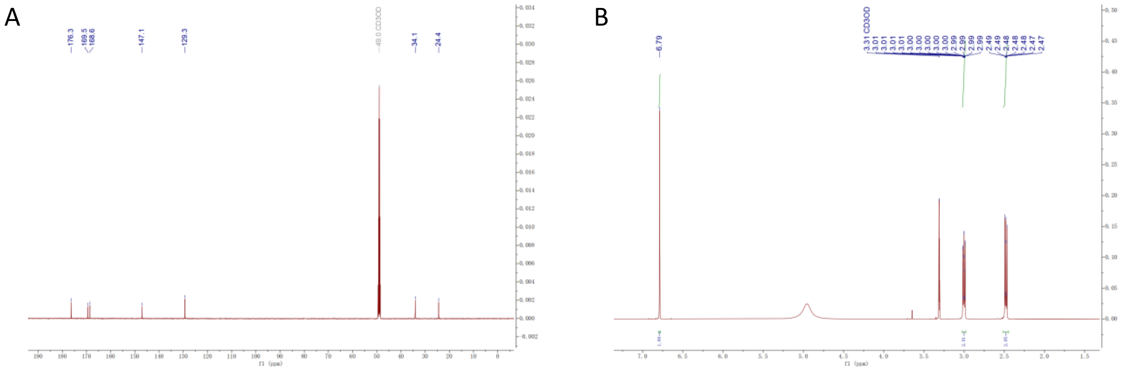

Figure S8. The ^{13}C NMR and ^{1}H NMR spectra of compound **5**. (**A**) ^{13}C NMR spectrum; (**B**) ^{1}H NMR spectrum

A B
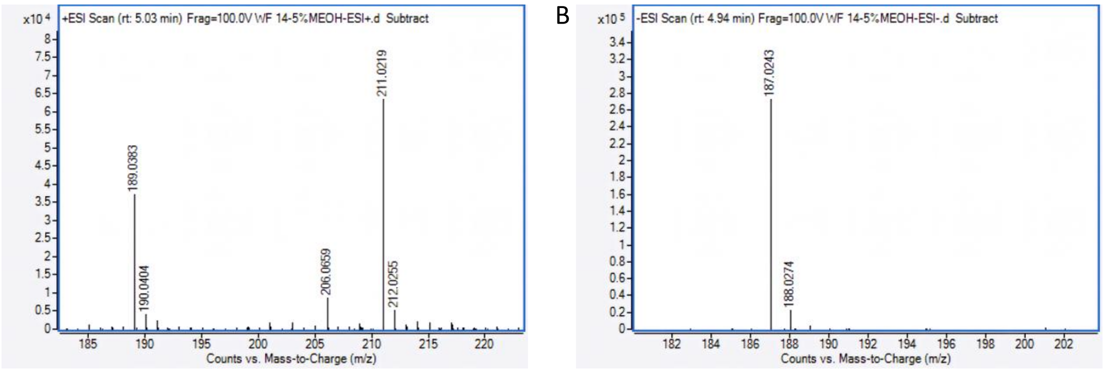

Figure S9. High-resolution ESI-MS spectra of compound **5**. (**A**) Positive ion mode; (**B**) Negative ion mode.

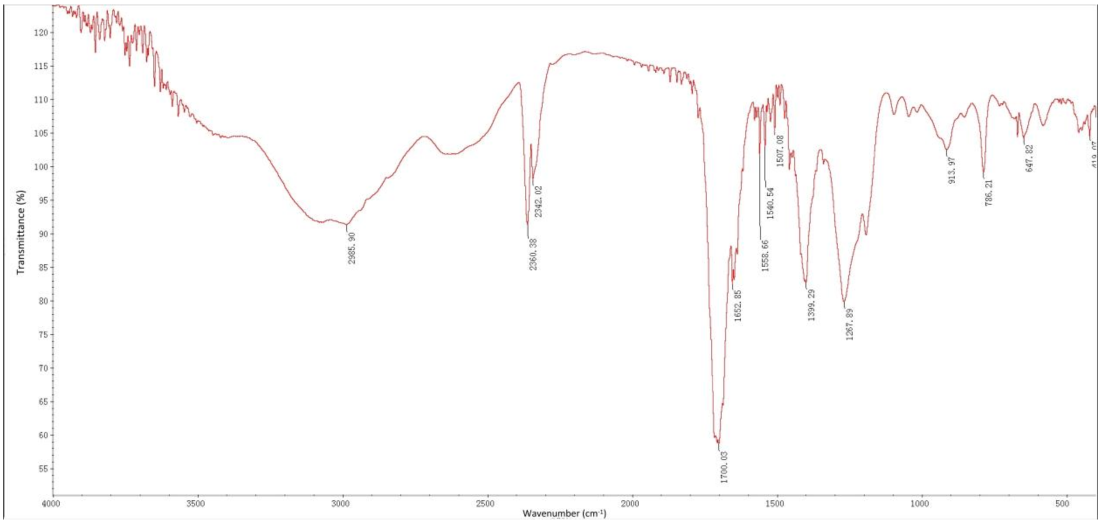

Figure S10. FT-IR spectrum of compound **5**. The spectrum was recorded in the wavenumber range of 4000–500 cm^{-1}, and the main characteristic absorption peaks are labeled with corresponding wavenumber values

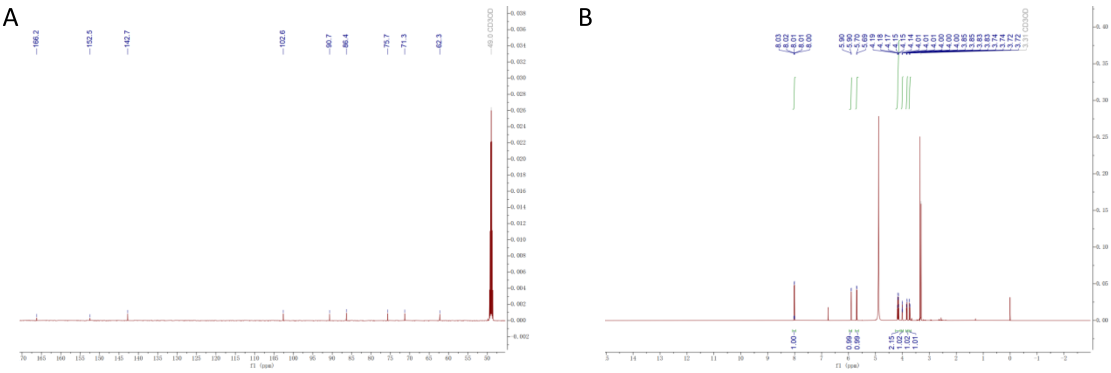

Figure S11. The ^{13}C NMR and ^{1}H NMR spectra of compound **6**. (**A**) ^{13}C NMR spectrum; (**B**) ^{1}H NMR spectrum.

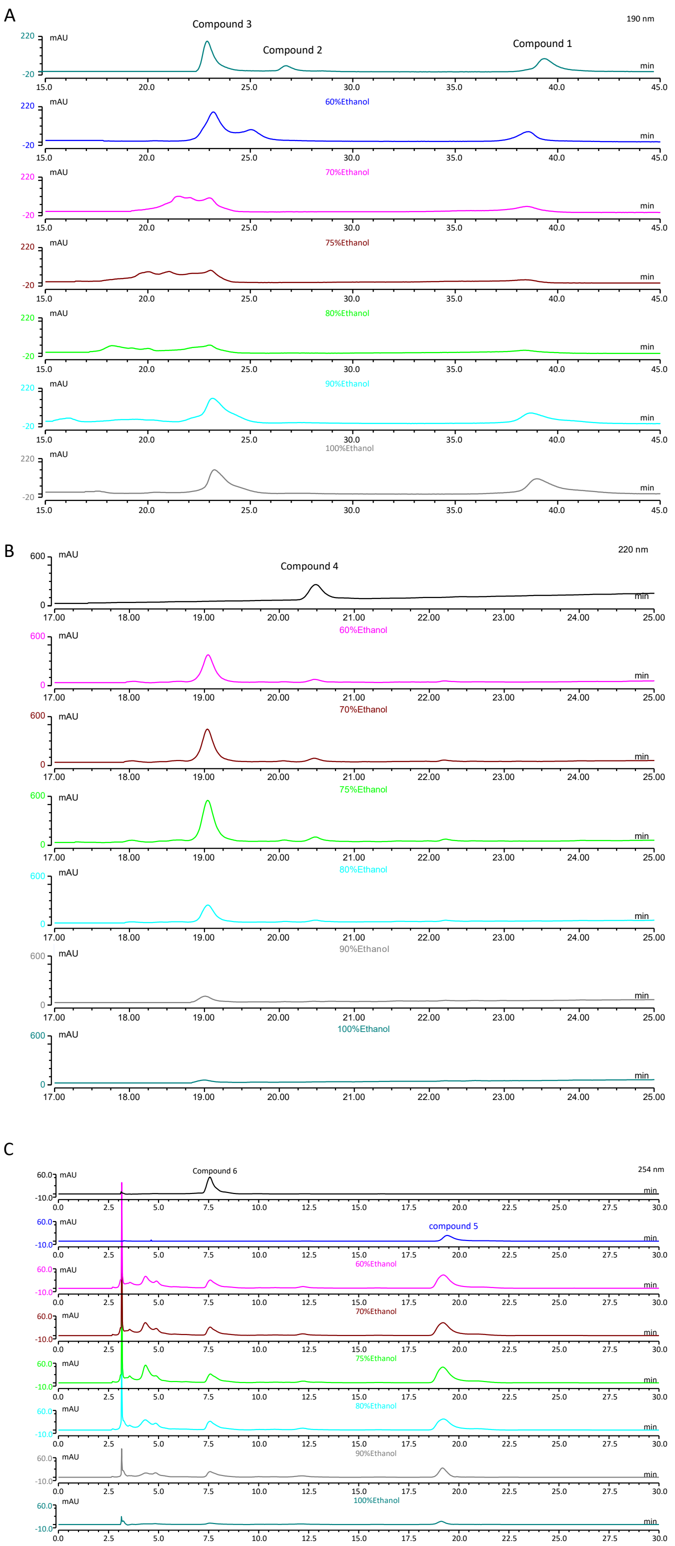

Figure S12. HPLC profiles of compounds **1**–**6** and crude extracts from different solvent extractions. (**A**) Compounds **1**-**3** and crude extracts. (**B**) Compound **4** and crude extracts. (**C**) Compounds **5**-**6** and crude extracts.

Table S1 HPLC purity of compounds **1**–**6**.

Compounds	Purity (%)
1	96.30
2	95.83
3	97.53
4	95.76
5	98.76
6	98.36

Table S2 Calibration curve equations and coefficients of determination (R^2) for compounds **1–6**.

**Compounds**	**Calibration curve equations**	R^2
1	Y=0.0993X+0.5483	0.9999
2	Y=0.4312X+0.6028	0.9994
3	Y=0.0750X-0.0123	0.9997
4	Y=1.6983X-1.2704	0.9999
5	Y=0.3757X-0.2183	0.9999
6	Y=0.8599X-1.8086	0.9956

Table S3 LOD and LOQ for compounds **1–6**.

Compounds	LOD (μg/mL)	LOQ (μg/mL)
1	0.42	1.39
2	1.81	6.05
3	0.80	2.67
4	8.71	29.04
5	3.41	11.35
6	5.64	18.81
